# Chromosome-scale genome assembly of *Rhododendron molle* provides insights into its evolution and terpenoid biosynthesis

**DOI:** 10.1186/s12870-022-03720-8

**Published:** 2022-07-15

**Authors:** Guo-Lin Zhou, Yong Li, Fei Pei, Ting Gong, Tian-Jiao Chen, Jing-Jing Chen, Jin-Ling Yang, Qi-Han Li, Shi-Shan Yu, Ping Zhu

**Affiliations:** 1 State Key Laboratory of Bioactive Substance and Function of Natural Medicines, NHC Key Laboratory of Biosynthesis of Natural Products, CAMS Key Laboratory of Enzyme and Biocatalysis of Natural Drugs, Institute of Materia Medica, Chinese Academy of Medical Sciences & Peking Union Medical College, 1 Xian Nong Tan Street, Beijing, 100050 China; 2 Institute of medical biology, Chinese Academy of Medical Sciences & Peking Union Medical College, 935 Jiaoling Street, Kunming, 650118 Yunnan Province China

**Keywords:** *Rhododendron molle*, Grayanoids, Genome, Terpene synthases, Cytochrome P450 monooxygenases

## Abstract

**Background:**

*Rhododendron molle* (Ericaceae) is a traditional Chinese medicine, which has been used to treat rheumatism and relieve pain since ancient times. The characteristic grayanoids of this plant have been demonstrated to be the chemical basis for the analgesic activity. Moreover, unlike morphine, these diterpenoids are non-addictive. Grayanoids mainly distribute in the leaves, flowers, roots, and fruits of *R. molle*, with low content. Currently the research on the biosynthesis of grayanoids is hindered, partially due to lack of the genomic information.

**Results:**

In the present study, a total of 744 Mb sequences were generated and assembled into 13 chromosomes. An ancient whole-genome duplication event (*Ad-β*) was discovered that occurred around 70 million years ago. Tandem and segmental gene duplications led to specific gene expansions in the terpene synthase and cytochrome P450 (CYP450) gene families. Two diterpene synthases were demonstrated to be responsible for the biosynthesis of 16α-hydroxy-ent-kaurane, the key precursor for grayanoids. Phylogenetic analysis revealed a species-specific bloom of the CYP71AU subfamily, which may involve the candidate CYP450s responsible for the biosynthesis of grayanoids. Additionally, three putative terpene biosynthetic gene clusters were found.

**Conclusions:**

We reported the first genome assembly of *R. molle* and investigated the molecular basis underpinning terpenoids biosynthesis. Our work provides a foundation for elucidating the complete biosynthetic pathway of grayanoids and studying the terpenoids diversity in *R. molle*.

**Supplementary Information:**

The online version contains supplementary material available at 10.1186/s12870-022-03720-8.

## Background

*Rhododendron* is the most diverse genus in the Ericaceae family and comprises over 1000 species. *Rhododendron molle* is a perennial shrub of the Ericaceae and is indigenous to southern areas China. In addition, *R. molle* has been used as an analgesic in traditional Chinese medicine and can be traced back to the earliest medicinal book, *Shennong Bencao Jing*. The dried flowers, roots and fruits of *R. molle* are primarily used to treat traumatic injury and rheumatism [1] . Additionally, the leaves of *R. molle* can be used as pesticides in agriculture [2]. Previous studies have shown that *R. molle* constitutes a remarkable source of secondary metabolites [1] . In particular, the characteristic grayanane diterpenoids of this plant have been highlighted for their significant analgesic activity [3–5]. Pharmacological studies have demonstrated that grayanoids can alleviate acute, inflammatory, and neuropathic pain; unlike morphine, grayanoids are non-addictive [3]. Despite their potential uses, the biosynthetic pathway of grayanoid is poorly understood, partially due to a lack of genomic information.

Grayanoids are tetracyclic diterpenoids. They have a distinct 5–7–6-5 ring system and are highly hydroxylated. In plants, the biosynthesis of terpenoids is initiated by terpene synthases (TPSs), which generate different kinds of terpene scaffolds. According to the phylogenetic relationships, these TPSs group into a mid-size family that is further divided into seven subfamilies, namely, TPS-a, TPS-b, TPS-c, TPS-d, TPS-e/f, TPS-g and TPS-h [6]. In angiosperms, the common precursor geranylgeranyl diphosphate (GGPP) is typically cyclized into different diterpene backbones via the sequential action of class II diterpene synthases and class I diterpene synthases. Diterpene backbones are further modified by cytochrome P450 monooxygenases (CYPs) and transferases to form the final functionalized diterpenoids. CYPs are the most important decorating enzymes and the major driver of diterpenoid diversity. In plants, CYPs represent one of the largest multigene families, accounting for approximately 1% of the protein-coding genes, and can be divided into ten clans. The CYPs involved in the biosynthesis of plant diterpenoids tend to be limited to a few clans, among which the CYP71 clan and CYP72 clan are mainly distributed in angiosperms. With recent improvements in the quality and cost-effectiveness, sequencing of the genome has become an indispensable tool for studying plant natural products in unexplored non-model species [7, 8]. Recently, the genomes of *Rhododendron simsii*, *Rhododendron williamsianum* and *Rhododendron delavayi* [9–11] were sequenced. Studies on these genomes have provided insight into the genome evolution and the mechanism of flower color formation, However, specialized secondary metabolites have not received attention in these species.

In the present study, a high-quality chromosome-scale assembly of the *R. molle* genome was presented for the first time. In total, 744.36 Mb of genome sequences were assembled, 472.24 Mb of which were repetitive sequences. A total of 94.57% of the assembled genome was sorted into 13 chromosomes. We discovered the remnants of an ancient whole-genome duplication (WGD) event in the *R. molle* genome. Additionally, the significant expansion of gene families involved in secondary metabolites was observed, especially terpene synthase (TPS) and cytochrome P450 (CYP) families. We identified two diterpene synthases (diTPSs) responsible for the biosynthesis of 16α-hydroxy-ent-kaurane, the key precursor of grayanoids. A species-specific bloom occurred within the CYP71AU subfamily, which is most likely associated with grayanoid biosynthesis. In addition, we also discovered three putative terpene biosynthetic gene clusters in the genome of *R. molle*.

## Results

### *R. molle* genome assembly and annotation

**Fig. 1:**
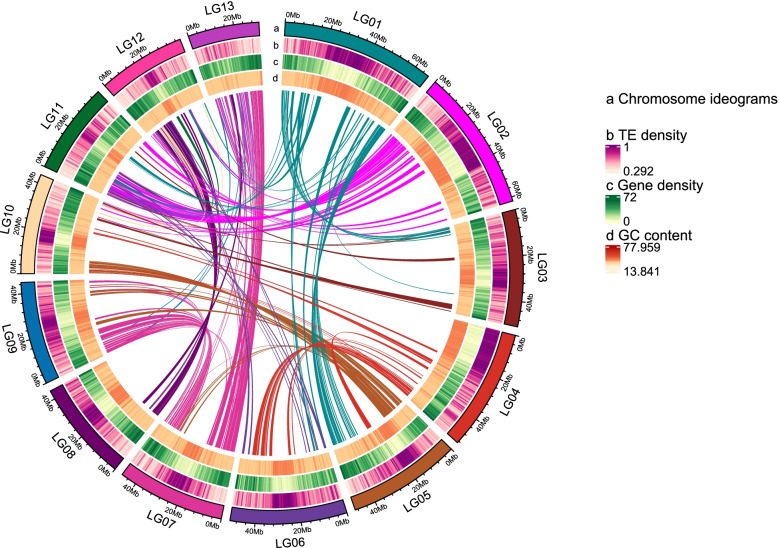
Overview of *R. molle* genome assembly features. The outer track represents chromosomes. Tracks from b to d represent the distribution of TE density, gene density and GC content, respectively. The inner circle represents syntenic gene pairs within *R. molle* genome. All distributions are drawn in a window size of 500 kb

A high-quality assembly of the *R. molle* genome was generated by combining Illumina short-read sequencing, PacBio SMRT (single-molecule real-time) sequencing, and Hi-C (high-throughput chromosome conformation capture) technology. Based on *k-mer* analysis, the genome size of *R. molle* was estimated to be 700.18 Mb with a high level of repeats (53.17%) and a relatively low level of heterozygosity (0.44%) (Additional file 1: Fig. S1 and Additional file 2: Table S1). 744.36- Mb sequences of the *R. molle* genome were assembled using 49.48 Gb PacBio long reads (66 × coverage) and 58.43 Gb Illumina short reads (83.45 × coverage) (Additional file 2: Tables S2-S3). In order to refine and obtain a chromosome-scale assembly, a Hi-C library was further constructed, and 703.95 Mb (94.57%) of the assembled genome was anchored into 13 chromosomes (Fig. 1 and Additional file 1: Fig. S2), among which 620.89 Mb of sequences could be ordered and oriented, accounting for 88.2% of the chromosome sequences (Additional file 2: Tables S1 and S4). To assess the quality of the genome assembly, we mapped Illumina short reads to it, and the mapping ratio was above 90%, indicating that most of the *R. molle* genome was assembled (Additional file 2: Table S5). BUSCO (Benchmarking Universal Single-Copy Orthologs) analysis showed that 90.90% of the gene sets were complete (1309 out of 1440 total lineage BUSCOs) (Additional file 2: Table S6). CEGMA (Core Eukaryotic Genes Mapping Approach) analysis revealed that our assembly contained 95.56% of highly conserved genes (237 out of 248 genes) (Additional file 2: Table S7).

The *R. molle* genome was annotated using a combined approach, including ab initio predictions, homology-based predictions, and RNA-seq based predictions (Additional file 2: Table S8). A total of 39,288 gene models were predicted. The gene density of the genome was around 53 genes/Mb, and 32,041 genes (81.55%) were found on the assembled chromosomes. Among these predicted genes, 36,678 (93.36%) were supported by both transcriptome data and ab initio predictions, indicating high accuracy of gene predictions. The average length of the genes is 4752 bp, with a mean intron length of 3146 bp and an average exon length of 1605 bp (Additional file 2: Table S9). Based on the gene prediction results, it was estimated that only 6.4% of the assembled *R. molle* genome was comprised of coding sequences. 88.43% of predicted genes were annotated by five public databases including NR, KOG, GO, KEGG and TrEMBL (Additional file 2: Table S10 and Additional file 1: Fig. S3). Among all annotated genes, 1925 genes were identified as transcription factors (TFs), including *AP2* (141), *bHLH* (136), *MYB* (154), and *WRKY* (65) families (Additional file 1: Fig. S3). We also performed similarity searches and annotated non-coding RNA (ncRNA) genes, yielding a total of 2628 ncRNA genes, including 103 microRNA (miRNA) genes, 667 transfer RNA (tRNA) genes, 288 ribosomal RNA (rRNA) genes, 1340 small nucleolar RNA (snoRNA) genes and 230 small nuclear RNA (snRNA) genes (Additional file 2: Table S11).

A total of 472 Mb repetitive sequences (63.52% of the assembly) were identified by integrated approaches including homology-based and de novo prediction (Additional file 2: Table S12). The level of repetitive sequences found in our assembly was higher than that in *R. simsii* (251 Mb, 47.48%) [11] (Table 1), indicating that the larger size of the *R. molle* genome may be attributed to the higher proportion of repetitive sequences. LTRs (long terminal repeats) are the most abundant repetitive elements, accounting for 36.86% of the total genome sequences. Most of the LTRs were Gypsy (29.05%) and Copia (5.21%) elements (Additional file 2: Table S12 and Additional file 1: Fig. S4). The ratio of Gypsy to Copia (5.57) was higher in the *R. molle* genome than that in other close relatives, such as *R. delavayi* (3.7) [9] and *R. simsii* (2.97) [11]. It is suggested that Gypsy transposon amplification is a key driver for the evolution of the *R. molle* genome. Furthermore, we discovered that intact LTR insertion events in *R. molle* genome were a continual process, with most insertions having occurred in the last 2 million years. We also observed similar continuous trends of LTR insertion in *R. simsii*, *R. delavayi*, *R. williamsianum* and their ancestor kiwifruit, suggesting that this phenomenon may be common in *Rhododendron* (Additional file 1: Fig. S5). In addition, a total of 956 simple sequence repeats were identified, which will provide useful molecular markers for *R. molle* breeding (Additional file 2: Table S12).

**Table 1:** Summary of the *R. molle* assembled genome and comparison with three other *Rhododendron* species

Assembly feature	***R. molle***	***R. delavayi***	***R. williamsianum***	***R. simsii***
Estimate of genome size (Mb)	700.18	*	525	*
Assembly genome length (Mb)	744.36	695.1	532.1	528.6
Repeat region in genome (Mb)	472	356	*	251
Contigs number	3581	*	*	911
Contigs N50 (kb)	836.61	61	*	2234
Longest Contigs (kb)	5461	581.4	*	*
GC content	41.15%	*	*	38.91%
Number of chromosomes	13	*	13	13
Length anchor on chromosomes (Mb)	703	*	368.4	481.9
Oriented percentage	88.2%	*	*	*
Scaffolds number	2537	193,091	11,985	552
Scaffolds N50 (Mb)	47.02	0.64	0.22	36.3
Scaffold max (Mb)	66.75	*	*	*
**Genome Annotation**				
Predicted gene models	39,288	*	*	34,170
Gene density/Mb	53	*	*	*
Average gene length (bp)	4752.73	4434.22	4628	5089.22
Average coding sequence length (bp)	1213.78	1153.21	*	1288.73
Non coding RNAs	2628	*	*	1171
Pseudogenes	6180	*	*	*

Asterisk (*) represent genomic data were not displayed in the original papers

### Evolution of the *R. molle* genome

**Fig. 2:**
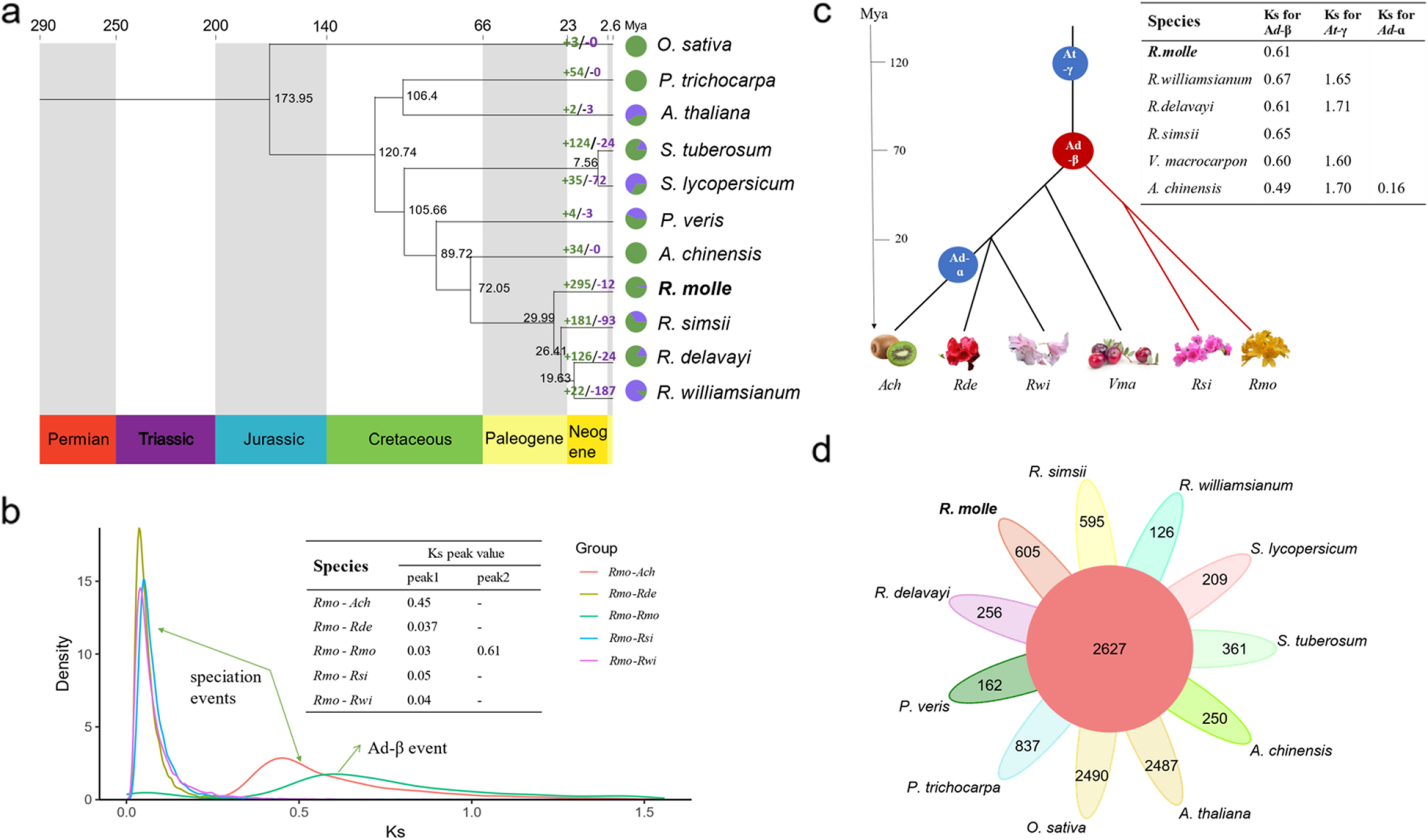
The evolution of *R. molle* genome (**a**) Phylogenetic relationship and estimated divergence time among eleven plant species. The phylogenetic tree was constructed using 1827 single-copy genes shared by all species used in current study, and the estimated divergence times are displayed by the black number at each branch node. **b** The distributions of the *Ks* values for paralogous genes in *R. molle* genome and orthologous genes from four other relative species. Abbreviation: *Rmo-R. molle*; *Ath*-*A. chinensis*; *Rde*-*R. delavayi*; *Rsi*-*R. simsii*; *Rwi*-*R. williamsianum*. **c** Whole-genome duplication events detected in *R. molle* and other Ericaceae species. **d** Petal diagram displaying the common and specific gene families between *R. molle* and ten other plant species. The number in middle circle represents the number of shared gene families among all species, and number in the edge represents the number of specific gene families in each species

The evolutionary history of *R. molle* was investigated based on its assembled and annotated genome. A set of 1827 single-copy orthologs from 11 angiosperm species was identified using OrthoFinder. Based on the sequence alignment of these single-copy orthologs, a phylogenetic tree was constructed, *Oryza. sativa* was assigned as an outgroup. The accuracy of the phylogenetic tree was supported by bootstrap values with a minimum support rate of 94 (Additional file 1: Fig. S6). This phylogenetic tree showed that *R. molle* was clustered with the other three *Rhododendron* species (*R. simsii, R. williamsianum* and *R. delavayi*) as expected, while this group diverged from their most recent common ancestor *A. chinensis*, approximately 72 million years ago (Mya). The phylogenetic relationship within the *Rhododendron* clade is consistent with previous studies, which demonstrated that *R. simsii* split from *R. molle* around 30 Mya (Fig. 2a). Whole genome duplication (WGD) frequently occurs in the genomes of land plants, and serves as an invaluable source of raw materials for gene genesis, neo-functionalization and sub-functionalization [12, 13]. We analyzed the synteny within the *R. molle* genome using MCScanX [14], and found 162 syntenic blocks throughout the whole genome, containing 1228 syntenic gene pairs (Additional file 2:Table S13). For paralogous gene pairs in syntenic blocks, only 47 pairs (3.5%) were located intra-chromosomally, while the rest were distributed inter-chromosomally (Additional file 1: Fig. S7). The syntenic depth among chromosomes was investigated to explore the number of WGD events. Among all syntenic genes within the *R. molle* genome, the majority (92%) have only one homolog on other chromosomes (Additional file 2: Table S13), implying a 1:1 syntenic relationship among chromosomes. These findings strongly indicated that a single WGD event occurred in the *R. molle* genome.

We further analyzed the synonymous substitutions per synonymous site (*Ks*) distribution of paralogous gene pairs throughout the entire *R. molle* genome. A signature peak at approximately 0.61 (Fig. 2b) indicates an ancient WGD event (referred to as *Ad-β*) occurred around 70 Mya, which is consistent with our earlier syntenic analysis. Additionally, a minor peak at around 0.03 was detected, which likely represent background small-scale gene duplication rather than recent WGD. The 4DTv (four-fold degenerate synonymous third-codon transversion rate) value was calculated. A major peak was detected at 0.24, which further supported the *Ad-β* event (Additional file 1: Fig. S8). In order to explore the number and timing of WGD events in the genome of Ericaceae species, we compared the *Ks* distributions in our study to those generated for other Ericaceae species in previous research [9–11]. Although lineages tend to have different molecular evolution rates, a peak at around 0.6 was found in all four Ericaceae genomes, indicating they all shared an ancient WGD event. This peak also corresponded to the *Ad-β* event identified in *A. chinensis* [15] (Fig. 2c). There was only a single major *Ks* peak for *R. molle* and *R. simsii*, which indicated that they have not experienced the *At-γ* event shared by most core eudicots, including *R. williamsianum* and *R. delavayi*.

### Gene family evolution related to terpenoids biosynthesis

The evolution of gene families was investigated by comparing orthologous genes between *R. molle* and ten other sequenced angiosperm species. Based on orthologous clustering analysis, a total of 41,197 orthologous gene families were identified, which comprised 271,568 genes (Additional file 1: Fig. S9). Among them, 2627 gene families were common to all eleven species, and 605 gene families that comprised 1714 genes were unique to *R. molle* (Fig. 2d and Additional file 2: Table S14). Functional enrichment analysis of *R. molle* unique genes using GO terms revealed that many unique genes were grouped into the metabolic process (GO:0008152) and catalytic activity (GO:0003824) (Additional file 1: Fig. S10 and Additional file 3: Table S15). It is indicated that possible species-specific biosynthesis processes lead to unique secondary metabolites.

The expansion or contraction of gene families in *R. molle* was explored using CAFÉ [16], The results revealed that 295 gene families expanded after divergence from *A. chinensis*, whereas only 12 gene families contracted (Fig. 2a and Additional file 1: Fig. S11). Functional annotation revealed that numerous genes were enriched in two critical families (TPS and cytochrome P450) involved in secondary metabolite biosynthesis, supporting that terpenoids are important specialized metabolites of this medicinal plant. Moreover, expanded gene families in *R. molle* were observed to be enriched in several KEGG pathways, including those associated with specific secondary metabolism, such as “phenylpropanoid biosynthesis”, “sesquiterpenoid and triterpenoid biosynthesis”, “diterpenoid biosynthesis” and “anthocyanin biosynthesis” (Additional file 1: Fig.S12 and Additional file 3: Table S16). The expanded TPS and CYP families may contribute to terpenoids diversity in *R. molle*.

### Genome mining for terpene synthase genes

**Fig. 3:**
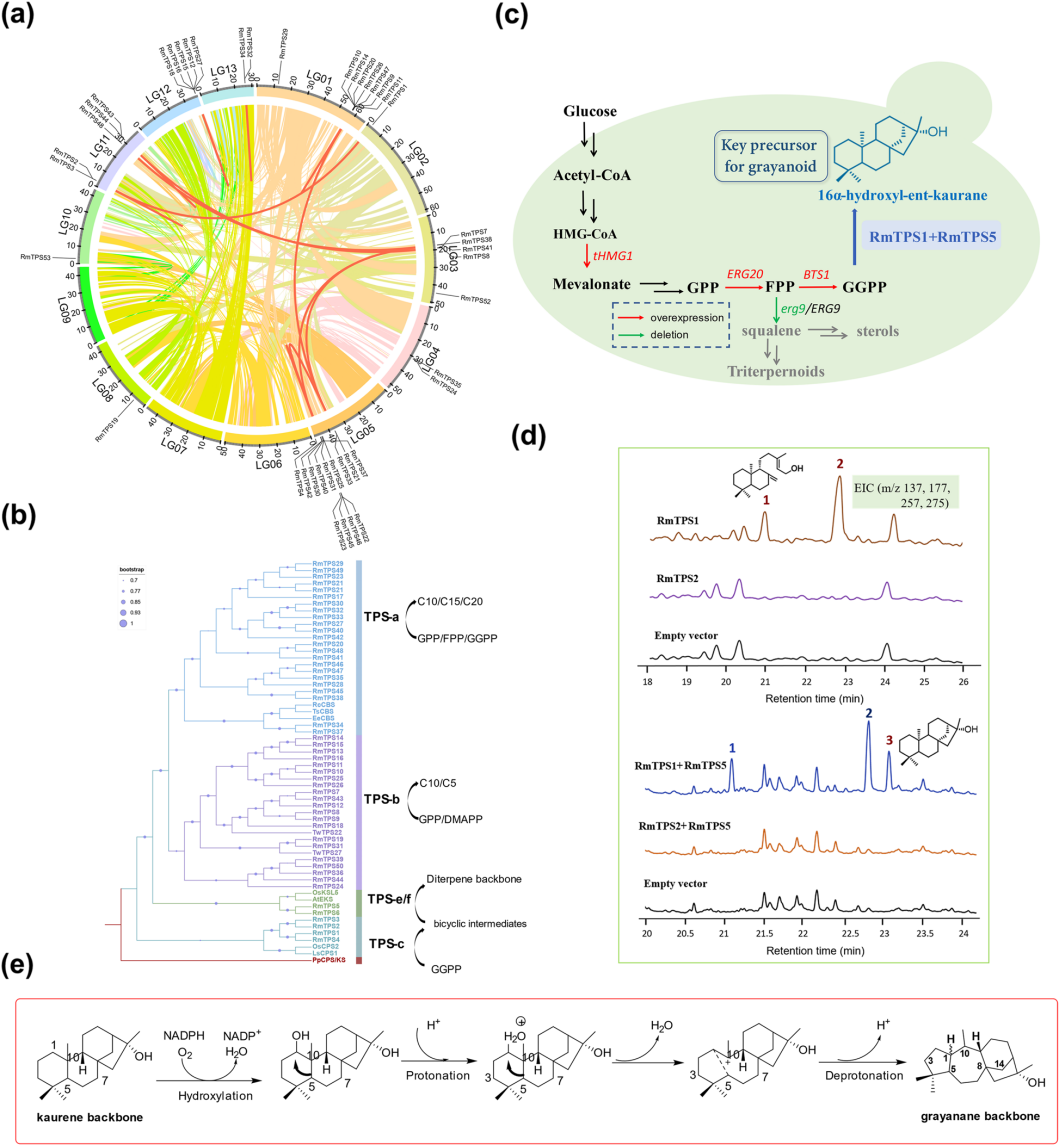
Terpene synthases in *R. molle* genome and functional characterization of diterpene synthases. **a** The circos map displaying chromosomal localization of all TPSs in *R. molle* genome. Tandem duplication genes are presented by short red lines, and the inter-chromosomal segmental duplication gene pairs are connected by red lines. The colour lines in background indicate all syntenic blocks in the genome of *R. molle*. **b** Phylogenetic analysis of total 50 putative terpene synthases *R. molle* genome, PpCPS/KS (*P. patens* copalyl diphosphate synthase /kaurane synthase) was assigned as tree root. Bootstrap values greater than or equal to 70% are shown. **c** Construction of an engineered yeast strain for production of diterpene. To increase the production of GGPP, a single allele of *ERG9* was knockout, *tHMG1*, *ERG20* and *BTS1* were overexpressed. **d** GC-MS analysis of yeast strains expressing RmTPS1, RmTPS2, respectively, two products were yielded in yeast strain harbouring RmTPS1, product 1 was identified as ent-copalol, the fragmentation pattern of product 2 matched with labd-13-en-8,15-diol in NIST database. GC-MS analysis of yeast strains combinational expressing of RmTPS1 + RmTPS5 and RmTPS2 + RmTPS5, 16α-hydroxy-ent-kaurane (product 3) was detected as a single new product of combinational expressing of RmTPS1 and RmTPS5. **e** The speculated rearrangement process from kaurane backbone to grayanane backbone. The process is involved a Wagner-Meerwein rearrangement, with the C5-C10 bond shifting to C5-C1 bond

TPSs are the key enzymes involved in the biosynthesis of terpenoids and catalyze the common isoprene precursors to the terpene backbone. A total of 50 TPSs were identified, with a gene density of 0.067 genes/Mb in *R. molle* genome (Additional file 2: Table S17). A total of 13 tandem duplicated gene pairs and 6 segmental duplicated gene pairs were detected among 50 TPS genes (Additional file 2: Table S18), indicating that the expansion of the TPS family is caused by these two events (Fig. 3a). Most TPSs are from the TPS-a and TPS-b families, according to the phylogenetic analysis (Fig. 3b). The chromosomal locations of members from these two families are characterized by massive tandem duplicated gene clusters (Fig. 3a and Additional file 2: Table S19). On chromosome 5, six TPS genes from the TPS-a family occurred in a 710-kb long cluster, and another two TPS-a genes with 89% identity were also located on chromosome 5 as a small cluster. In addition, five TPS-a members were densely clustered together on chromosome 12 in a stretch of 80 kb. Members of the TPS-b family were mainly distributed in three tandem clusters. Seven genes were found on chromosome 1 in a tandem array across a stretch of 458 kb. The other two clusters were located on chromosome 11 and chromosome 5 and occurred in the stretch of 24 kb and 48 kb, respectively, each of which contained two genes.

Among the 50 TPS candidates, there were four CPSs (CPP synthases, class II TPSs), one KS (kaurane synthase, class I TPSs) and one putative KSL (kaurane synthase-like). The four CPS candidates contained the aspartate-rich DxDD motif in their N-terminal, while KS and KSL candidates harboured characteristic DDxxD motifs in their C-terminal (Additional file 1: Fig. S13). The class II TPS (CPS) and class I TPS (KS) could consecutively catalyze GGPP to form the diterpene backbone [17]. Phylogenetic analysis revealed that CPS candidates (*RmTPS1–4*) and KS candidates (*RmTPS5* and *RmTPS6*) were grouped into TPS-c and TPS-e/f families, respectively (Fig. 3b and Additional file 1: Fig. S13). They were putative candidates involved in kaurane biosynthesis. Masutani et al. [18] fed the leaf of *Leucothoe grayana* Max with ^14^C-labeled en-kaurane. After 12 days, ^14^C-labeled grayanotoxin III was isolated from the leaf, indicating ent-kaurane could be a pivotal precursor involved in the production of grayanotoxin. Hanson [19] proposed that the kaurane skeleton is the parent backbone of grayanane and can be converted to grayanane via oxidative rearrangement.

### Functional characterization of diterpene synthases

We used yeast (*Saccharomyces. cerevisiae*) to test the biochemical functions of TPS candidates. An engineered yeast strain was first constructed to increase the supply of GGPP precursor (Fig. 3c). In the endogenous yeast MVA pathway, *ERG9* acts downstream of *ERG20*, consumes FPP as its substrate, leading to the synthesis of squalene, which would decrease FPP flux flow towards GGPP biosynthesis. To accumulate sufficient FPP for conversion to GGPP, we constructed a single allele deletion for *ERG9* in the diploid yeast strain INVSc1 (Additional file 1: Fig. S14), which would ensure the robustness of the yeast strain and increase FPP accumulation, hence improving GGPP production in the yeast cell indirectly. Ignea et al [20] took a similar approach to enhance terpenoid production in yeast. We employed a loxP-KanMX-loxP deletion cassette to delete one of the two alleles in the INVSc1 strain, and generate the *ERG9* haploid deficient mutant yeast strain IN-0. Another key regulatory point in the MVA pathway is the conversion of HMG-CoA (hydroxy-3-methylglutaryl coenzyme A) into mevalonate [21], catalyzed by two HMG-CoA reductases (HMG1 and HMG2), which are encoded by *hmg1p* and *hmg2p*, respectively [22]. HMG1 is much more stable than HMG2 and contributes most of the activity [22], making it the rate-limiting enzyme in the MVA pathway. Previous studies have demonstrated that overexpression of the catalytic domain of HMG1 (tHMG1) can result in increased production of terpenoids [23]. The work by Zhou et al. proved that the fusion of BTS1-ERG20 can channel the FPP flux to diterpene production, thereby reducing its consumption via other pathways [24]. We ligated the BTS1-ERG20 construct into the high-copy yeast expression plasmid pESC-LEU, along with tHMG1, and the recombinant plasmid pESC-LEU-(BTS1-ERG20)-tHMG1 was introduced into yeast strain IN-0 to generate strain IN-1, which was used as chassis to examine enzymatic function of the diterpene synthase. In our previous transcriptome studies of *R. molle*, three diterpene synthases (ID in RNA-seq: c95076, c86861 and c92044) were identified [25], corresponding to *RmTPS1*, *RmTPS2* and *RmTPS5*, respectively. The full-length cDNA sequences of these genes were obtained by 5′- and 3′-RACE and cloned into the yeast expression vector pESC-URA individually or in combination. *RmTPS1* and *RmTPS2* were introduced into the engineered yeast strain IN-1, resulting in strain IN-2 and IN-3. Expression of *RmTPS1* resulted in the production of two compounds (termed as product **1** and product **2**), and the GC-MS analysis revealed their retention time were at 22 and 22.8 min, respectively (Fig. 3d). The fragmentation pattern of product **1** was compared with that in the NIST database (Additional file 1: Fig. S15), and its structure was inferred to be ent-copalol, which was further confirmed by NMR (Additional file 1: Fig. S16 -S17). This result indicated that *RmTPS1* was a functional class II diTPS, and can convert GGPP into ent-copalyl diphosphate (ent-CPP), which was dephosphorylated into ent-copalol by yeast endogenous phosphatases. The characteristic mass ions of Product **2** were m/z 275, 257, 177, and 137. By comparison with the NIST database, it was identified as labd-13-en-8,15-diol (Additional file 1: Fig. S15). The formation of the oxygenated product resulted from water quenching at the C-8 carbocation prior to deprotonation. However, no product was detected in the yeast strain carrying either RmTPS2 or the empty pESC-URA vector. We further transformed the constructs pESC-URA-RmTPS2-RmTPS5 and pESC-URA-RmTPS1-RmTPS5 into the yeast strain IN-1, creating strains IN-4 and IN-5 (Additional file 2: Table S20). The yeast strain carrying pESC-URA-RmTPS1 or empty vector was used as control. Only co-expression of *RmTPS1* and *RmTPS5* afforded 16α-hydroxy-ent-kaurane as the single product (Fig. 3d). These results indicated that *RmTPS5* possessed class I diTPS activity and catalyzed ent-CPP that derived from GGPP by RmTPS1 to form 16α-hydroxy-ent-kaurane (Additional file 1: Fig. S18-S19). The majority of grayanoid compounds isolated from *R. molle* have an α-hydroxy groups at C-16 (Additional file 1: Fig. S20). It is suggested that 16α-hydroxy-ent-kaurane is a key intermediate in the biosynthetic pathway of grayanoids, which can be converted into the unique 5–7–6-5 tetracyclic grayanane backbone via oxidation-induced bond rearrangement (Fig. 3e).

### Chromosomal distribution and evolution of RmCYP genes

**Fig. 4:**
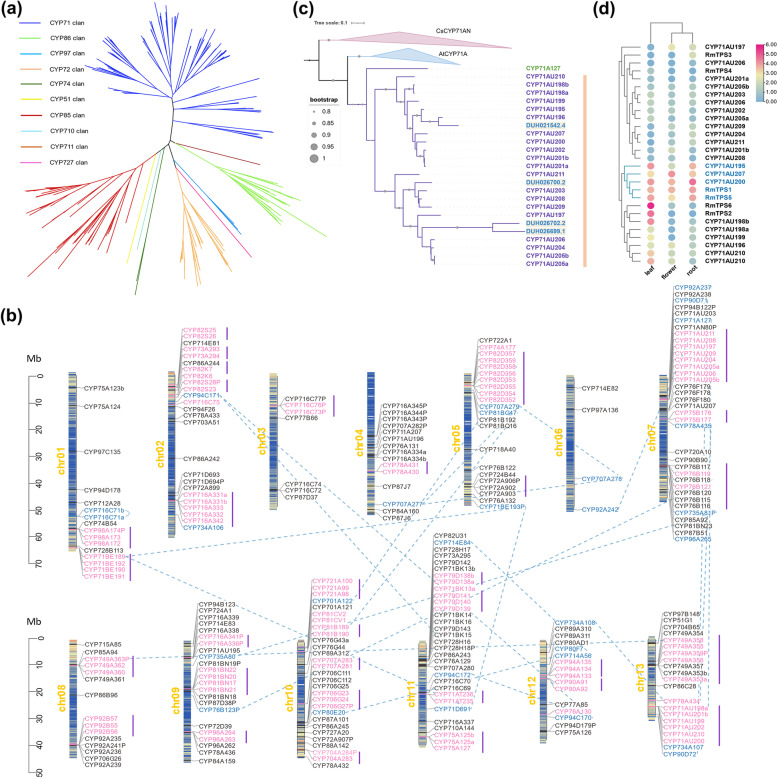
Analysis of CYP candidates. **a** The unrooted maximum likelihood phylogenetic tree show that all RmCYPs can be divided into ten clan, each clan were presented by different colour branches. **b** The bar plot showing the chromosomal distribution of RmCYPs. The serial number of each chromosome are showed to the left of bars, and the colour line within each bar represent gene density. The tandem duplication genes within chromosomes and the inter-chromosomal segmental duplication gene pairs are indicated in pink and blue, respectively. The CYP gene clusters are displayed using violet vertical lines, and segmental duplication gene pairs are connected by blue dashed lines. **c** Maximum likelihood (ML) tree of members from CYP71 family showing a specific-bloom of CYP71AU subfamily in *R. molle*. Gary circles represent bootstrap value larger than or equal to 0.8. Genes in gray background were candidates from *R. delavayi*. **d** The heatmap illustrating the co-expression pattern of CYP71AUs with RmTPSs

Given that cytochrome P450 monooxygenases (CYPs) play a significant role in terpenoid biosynthesis, we performed the genome-wide analysis of CYP candidates in *R. molle*, and identified a total of 294 CYP genes. An unrooted maximum likelihood (ML) tree was constructed based on the protein sequences of all RmCYP genes and *A. thaliana* CYP450 (AtCYPs) (Additional file 1: Fig. S21). Phylogenetic analysis revealed that the CYP genes were divided into ten clans (CYP51, 71, 72, 74, 85, 86, 97, 710, 711 and 727), including 46 families (Fig. 4a and Additional file 3: Table S21). RmCYP genes were unevenly distributed across the 13 chromosomes of *R. molle*. Chromosome 7 contained the most CYP genes (41) with gene density 0.78 gene/Mb, while chromosome 6 had the least number (4) and the lowest gene density (0.07 gene/Mb). To further explore the evolution of the CYP family in *R. molle*, the duplication events of RmCYP genes were analyzed. We discovered 31 tandem duplicated gene clusters including 67 tandem duplicated gene pairs (Additional file 3: Table S22), accounting for more than half of the total RmCYP genes (Fig. 4b). These clusters are distributed on all chromosomes except chromosome 6, and most contain more than three genes (Fig. 4b). Moreover, 26 segmental duplication events involving RmCYPs were detected (Fig. 4b and Additional file 3: Table S22). A few RmCYP genes (*CYP71AU198a*, *CYP749A363P*, *CYP76B118*, and *CYP82D358*) have undergone both segmental and tandem duplication events. Duplication events occurred in CYP71, CYP82 and CYP716 families more often than others, indicating that they are the major contributors to the RmCYP family expansion. The *Ka/Ks* (ω) ratios of all duplicated gene pairs were calculated, ranging from 0.049 to 2.019 (Additional file 1: Fig. S22). Their corresponding duplication events were estimated to occur between 0.19 to 212.9 Mya (Additional file 3: Table S22). Most ω values are less than 0.5, only one segmental duplicated pair *CYP716C71b*/*CYP716C71a* has a ω value greater than one (Additional file 1: Fig. S22), implying that the majority of genes are under purifying selection after their duplication events.

### Species-specific bloom of CYP71AU subfamily in *R. molle* genome

Among the large number of CYP genes, a species-specific bloom of the CYP71AU subfamily was identified in the *R. molle* genome by phylogenetic analysis (Fig. 4c). This subfamily belongs to the CYP71 clan and has been reported to be involved in terpenoid biosynthesis. The *R. molle* genome contains a total of 20 CYP71AU members, and the motifs and gene structures within this subfamily are relatively conserved (Additional file 1: Fig. S23). Among them, 12 tandem duplicated pairs were detected, and members in this expanded subfamily were organized in tandem arrays, indicating successive tandem duplication events led to the expansion of this subfamily. Eight CYP71AU genes formed the largest CYP cluster on chromosome 7, with a stretch of 257 kb (Fig. 4b). Three successive tandem duplicated genes were located on contig01509 as a small cluster, and six members were clustered together with two TPSs. The CYP71AU subfamily has expanded during evolution through multiple tandem duplication events, which is indicative of involvement in specialized grayanoids metabolism. The expression pattern of the CYP71AU subfamily members, along with five diterpene synthases in flower, leaf and root were further assessed. We observed that *CYP71AU200*, *CYP71AU207* and *CYP71AU195* were highly expressed in all three tissues and displayed similar expression profiles to *RmTPS1* and *RmTPS5* (Fig. 4d). Therefore, these three members from the CYP71AU subfamily were most likely to be the candidates for grayanoid biosynthesis.

### Discovery of terpenoid biosynthetic gene clusters

**Fig. 5:**
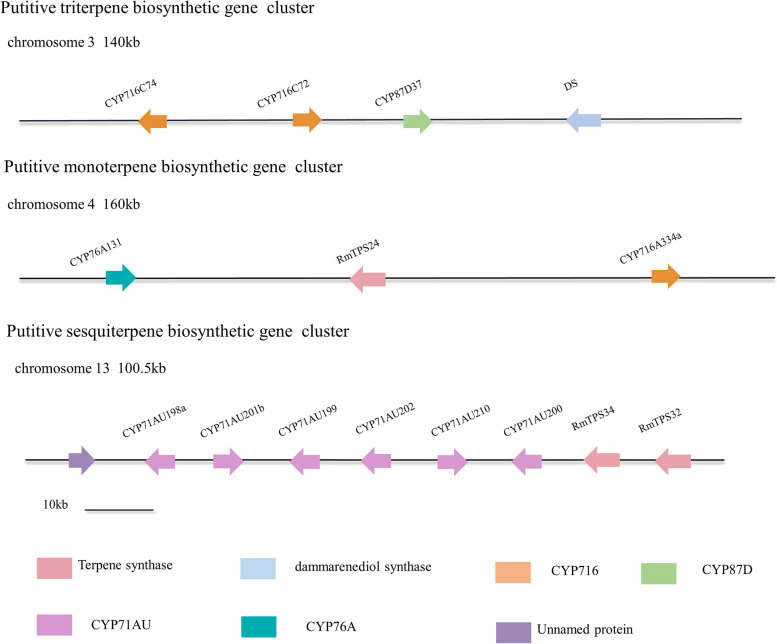
Discovery of terpene biosynthetic gene clusters in *R. molle* genome. The triterpene, monoterpene and sesquiterpene putative terpene biosynthetic gene clusters found in the genome of *R. molle*

With the rapidly growing number of available plant genomes, TPSs and CYPs in particular have been found to frequently cluster together in plant genomes and catalyze successive biosynthetic steps in terpenoid biosynthesis [26]. *R. molle* genome was analyzed to determine whether it contains potential biosynthetic gene clusters (BGCs) related to grayanoids biosynthesis. *RmTPS1* and *RmTPS5* were located separately and neither of them were flanked by CYP genes. However, our study revealed three other terpenoid putative BGCs (Fig. 5). A 160-kb genomic region on chromosome 4 contained one monoterpene synthase and two putative members from the CYP76A and CYP716 subfamilies. In addition, a 140-kb putative triterpene cluster was detected on chromosome 3, including one core scaffold-generating enzyme annotated as dammarenediol synthase, which was co-clustered together with two genes encoding enzymes from the CYP716C subfamily and one member from the CYP87D subfamily. It can be inferred that these CYPs were putatively involved in the oxidation of the initial triterpene hydrocarbon scaffolds produced by dammarenediol synthase. We also discovered that nine genes were organized as a relatively dense cluster in a 110-kb stretch on chromosome 13. This cluster contained two TPSs (*RmTPS32* and *RmTPS34*), which were a tandem duplicated pair with only three different amino acids, suggesting their biochemical function were identical to each other. *RmTPS32* and *RmTPS34* were duplicated at around 2.48 Mya, they represent a very recent TPS gene duplication. The two TPSs were flanked by six CYP paralogues from the CYP71AU subfamily and a gene of unknown function. The two TPSs belonged to the TPS-a family, implying the cluster might participate in sesquiterpenoid biosynthesis.

## Discussion

Plants are an important source of natural products, many of which can provide compounds for clinical use as pharmaceutical agents. As a traditional Chinese medicinal plant, *R. molle* has been exploited for its pain-relieving properties since ancient times. Modern phytochemistry studies revealed that *R. molle* contained abundant terpenoids, including tetracyclic grayanoids, which are the chemical basis for its analgesic activities [3, 5, 27]. To explore the biosynthesis of terpenoids, including grayanoids in particular, we employed Illumina sequencing in combination with PacBio sequencing and Hi-C approaches to obtain high quality assembly of *R. molle* genome. A total of 744.36 Mb genome sequence was generated with a contig N50 of 836.61 kb. Moreover, chromosome-level pseudomolecules have been constructed, the majority of the assembled sequences can be anchored onto 13 chromosomes. Hence, the integrated strategy used here proved to be highly effective in assembling the genome of *R. molle*. The availability of *R. molle* genome also allowed us to investigate its evolutionary history. The whole genome duplication event plays a significant role in plant genome evolution and adaptation to the local ecological niche. An ancient WGD event (*Ad-β*) in the genome of *R. molle* was detected. Previous comparative genomic analysis indicated that the *Ad-β* event was shared among Ericaceae lineages [10, 11], which has been further supported by our analysis of the *R. molle* genome. As more Ericaceae genomes become available, additional studies are needed to further explore the origin of this event. Analysis of *R. molle* genome revealed more gene families experiencing expansion. Especially many gene families involved in secondary metabolite biosynthesis were found to be expanded, including the TPS family and the CYP family, with expansions that occurred via tandem and segmental duplication events. The expansions likely play a significant role in the production of specific terpenoids in *R. molle,* which is the result of adaptation to the circumstance through long evolutionary process.

We explored the genetic basis underlying terpenoid biosynthesis, with a focus on analyzing related gene families, to lay a foundation for understanding the grayanoid biosynthetic pathway. A total of 50 TPSs were identified in this study, five of which were diterpene synthases, while other terpene synthases were from TPS-a or TPS-b families, which implied that sesquiterpene and monoterpene metabolites in *R. molle* are highly diverse. A remarkable feature of TPS gene arrangement on chromosomes is organized as tandem clusters, which is the consequence of local gene tandem duplication. All five diterpene synthases were annotated as CPS or KS/KSL. In combination with our previous transcriptome analysis, the biochemical functions of class II and class I diterpene synthase (*RmTPS1* and *RmTPS5*) were characterized. They successively catalyzed GGPP into 16α-hydroxy-ent-kaurane, an important biogenetic precursor that can be further rearranged into the grayanane backbone. This process was speculated to start with C1 hydroxylation of the kaurane backbone, whose hydroxyl group has been protonated. The protonated intermediate underwent water abstraction to produce an allylic carbocation, followed by a Wagner-Meerwein rearrangement, with the C5-C10 bond shifting to C5-C1 bond and forming a tertiary carbocation at C10. Finally, C10 was deprotonated, resulting in the characteristic 5–7–6-5 ring structure of grayanane (Fig. 3e).

It is difficult to elucidate the biosynthetic pathway of complex terpenoids, such as grayanoids. The main challenge remains to identify candidates involved in the downstream pathway of the terpene backbone from complex and large plant genomes. It is well known that CYPs are the major catalysts in the modification of the terpene backbone to form complicated terpenoids [28]. Our genome sequencing enables the identification of 294 members from the CYP super family, accounting for approximately 1% of the annotated genome. During evolution, the CYP genes from most species have undergone duplication and functional diversification [29]. In the current study, gene family expansion analysis was performed in combination with comparative phylogenetics to reveal a species-specific bloom that occurs in the CYP71AU subfamily. This subfamily has been demonstrated to play roles in the biosynthesis of terpenoids in angiosperms, and CYPs from the same subfamily are often associated with consecutive enzymatic reactions in biosynthetic pathways. Furthermore, three members in this subfamily were found to display similar expression patterns to *RmTPS1* and *RmTPS5*. Hence, the bloom detected in the *R. molle* genome plays a role in oxidizing the kaurane backbone and driving grayanoid diversity.

In recent years, it has been discovered that the biosynthetic pathways of secondary metabolites in plants are sometimes chromosomally clustered [30, 31], which can be applied to find new enzymes and accelerate the discovery and elucidation of biosynthetic pathways [32]. Unlike the BGCs found in bacteria and fungi, BGCs discovered in plants are much longer with more variable intergenic distances and often only contain part of the biosynthetic pathway of a metabolite [33]. The genome of *R. molle* was studied to explore putative BGCs, and a total of three putative terpene clusters were discovered. The BGCs located on chromosome 13 are of particular interest, and this cluster contains multiple P450 paralogues from the CYP71AU subfamily. These CYP paralogues may carry out successive oxidative decoration of the terpene backbone, leading to chemical diversification within the pathway. Further biochemical characterization of this cluster is necessary, which will elucidate how a pathway can gain complexity and elongation via tandem P450 gene duplications. Additionally, two other putative terpene clusters were discovered, and further biochemical characterization will be required to confirm their functions. The BGCs discovered in our study confirmed that TPS/CYP gene pairs are often clustered together in plant genomes, contributing to the current understanding of the metabolite diversity of *R. molle*.

## Conclusion

Our study not only represents a foundation for phylogenomic studies of *R. molle*, but also facilitates the evolutionary study of Ericaceae plants more generally. Our work primarily focuses on exploring the molecular basis underlying terpenoid biosynthesis. We have uncovered the diterpene synthases responsible for biosynthesis of the important precursor 16α-hydroxy-ent-kaurane, which is an important step towards elucidating the whole biosynthetic pathway of grayanoids. Through our genomic analysis, it is found that *R. molle* has the potential to produce a wide array of terpenoids, promoting the exploration of additional bioactive compounds. In addition, the genetic information provided by our study can be applied to the molecular breeding of *R. molle*, which will facilitate the conservation and sustainable utilization of this valuable species.

## Methods

### Plant material and DNA extraction

The *Rhododendron molle* cultivar used for genome sequencing was collected from the wild field in Guilin, Guangxi Province, China. No specific licenses or permissions from local government were required for our collection. The voucher specimen (ID-24757) was identified by doctor Guang-Zhao Li from Guangxi Institute of Botany and deposited in the herbarium at institute of Materia Medica, Chinese Academy of Medical Sciences (CAMS). This *R. molle* cultivar was cultivated at the greenhouse in the Institute of Medicinal Plant Development, CAMS. Fresh and healthy leaves were collected, and washed with tap water and ultrapure water three times to remove external contaminants. The cleaned leaves were flash-frozen using liquid nitrogen, and kept in − 80 °C for subsequent DNA extraction. The genomic DNA was extracted by CTAB method [34], and the protein and RNA contamination were removed using proteinase K and RNase A. To support genome annotation, the flowers, stems, leaves and roots were also collected for the preparation of RNA-seq (Additional file 4: method S1).

### Estimation of *R. molle* genome size

The genome size of *R. molle* was estimated by analyzing the *k-mer* (*k* = 21) frequency [35]. We generated 21*-mer* frequency information using paired-end reads from 350 bp library. The distribution of the 21*-mer* follows a Poisson’s distribution, and genome size was calculated with the eq. G = N/D, N represent the total *k*-*mers* number and D is average sequence depth. The total number of *k-mer* (*k* = 21) number was 47,474,641,778 after removing low-frequency *21-mer*, the average *k*-mer depth (67) is corresponding to the volume peak, hence the genome size was estimated to be 700.18 Mb.

### Library construction and genome sequencing

To construct Illumina library, the isolated genomic DNA was broken into random fragments. Quality control was performed using agarose gel electrophoresis and NanoDrop spectrophotometer. Paired-end sequencing library with 350 bp insert size was constructed based on the standard protocols from manufacturer (Illumina, San Diego, CA). The constructed library was sequenced by Illumina Novaseq 6000.

For pacbio libraries, 10 μg of genomic DNA was sheared to 20 kb by Covaris g-TUBE (Covaris, USA), then undergone purification and concentration using the pre-washed AMPure XP beads. The 20-kb template was prepared by BluePippin system (Sage Sciences). The sequencing libraries were subsequently constructed based on manufacturer’s instruction (Pacific Biosciences, CA, USA) and sequenced on a PacBio RSII platform.

Healthy young leaf from the same plant were fixed using formaldehyde to capture the interaction of DNA fragments, then the cross-linked DNA were cut by restriction enzyme, the sticky ends were biotinylated and ligated. The biotin-labelled products were purified, sheared and pulled down. Finally, the Hi-C libraries were constructed and sequence on Illumina platform with 150 bp paired-end reads.

### Genome assembly and annotation

De novo genome assembly was carried out using PacBio data. The subreads with length over 500 bp were selected and errors were corrected using Canu (v1.5.) [36]. The corrected reads were subsequently assembled into contigs by wtdbg [37]. Illumina paired-end reads were aligned into contigs to correct the original assembly using Pilon (v1.22) [38], which yielded total 744 Mb assembled genome with a contig N50 of 836.61Kb. Based on valid Hi-C data, 703 Mb sequences were anchored into 13 chromosomes using LACHESIS software [39].

To predict gene models in *R. molle* genome, we used an integrative approach, including de novo prediction, homology-based prediction and RNA-seq based prediction. De novo prediction was carried out using Genscan [40], Augustus (v2.4) [41], GlimmerHMM (v3.0.4) [42], GeneID (v1.4) [43] and SNAP (v2006-07-28) [44]. Protein sequences from *A. thaliana*, *A. chinensis* and *R. delavayi* were employed for homology-based prediction using GeMoMa (V1.3.1) [45]. In the RNA-seq based prediction approach, unigenes were first assembled using Stringtie (v1.2.3) [46] and Hisat (v2.0.4) [47], and gene models were predicted by PASA (v2.0.2) [48], GeneMarkS-T (v5.1) [49] and TransDecoder (v2.0) (http://transdecoder.github.io.) All the predicted gene models were integrated into a non-redundant gene set using EVM (v1.1.1) [50], which was further polished by PASA [48] to adjust exon boundary and untranslated regions. To annotate gene function, we performed similarity searches against the public databases NR, KOG, GO, TrEMBL and KEGG using BLAST (v2.2.31) [51].

Non-coding RNA was annotated using different strategies based on different structural features of them. The tRNA genes were identified using tRNAscan-SE software [52]. The rRNA and miRNA genes were predicted by whole genome alignment against the Rfam (http://rfam.xfam.org/) database using BLASTN [51].

Repetitive sequences were predicted by combination of homology-based and de novo-based approaches. We used LTR FINDER [53] and RepeatScout [54] to build repeat library. The library is classified by PASTEClassifier [55], and then combined with the RepBase database [56] to form the final repeated library. The repetitive sequences of in *R. molle* genome were predicted with RepeatMasker (v4.0.6) [57] based on the constructed final repeated library.

### LTR insertion time analysis

We used LTR Finder and PS scan to search for LTR sequences with scores value > 6 in the genome, and filter out the redundant sequences [58]. Multiple alignment of 5′- and 3′-LTR sequences was performed using MUSCLE [59]. The insertion time was calculated using the formula: T = K/2r, where K indicate the distance between alignment pairs, and r indicate base substitution rate. Distance K was calculated using Kimura model in EMBOSS (http://emboss.sourceforge.net/), and r value was set to 7 × 10^− 9^.

### Gene family expansion and contraction

The expanded and contracted gene families were identified using CAFÉ (v4.2) [16]. We used a birth-and-death model to evaluate gene gain or loss in gene families compared with their ancestors. Viterbi method was used to determine significant expansion or contraction of gene families. The family-wide *P*-values threshold and viterbi P-values threshold were set less than 0.05. The expansion and contraction gene families among all eleven species were annotated using PANTHER [60]. GO and KEGG enrichment for expanded and contracted gene families in *R. molle* genome were analyzed using clusterProfile [61].

### Phylogenetic tree and divergence estimation

To investigated the evolutionary history of *R. molle*, we performed comparative genome analysis of *R. molle* and ten other plant species (*A. thaliana*, *P. trichocarpa*, *S. lycopersicum*, *S. tuberosum*, *P. veris*, *R. simsii*, *R. williamsianum*, *R. delavayi*, *A. chinensis* and *O. sativa*). The OrthoFinder (v 2.4) [62] was used to find orthologous clusters in the given genomes. The orthologous genes were retrieved through sequence similarity search using diamond [63] with an E-value cutoff of 0.0001 and annotated using PANTHER (v15) database, where a minimum of 90.9% of the species have single-copy genes. The 1827 single-copy genes were subjected to multiple-sequence alignment with MAFFT v7.205 [64](−-localpair --maxiterate 1000) and the alignments were trimmed with Gblocks v0.91b(−b5 = h) [65]. We construct the maximum likelihood (ML) tree using IQ-TREE (v1.6.11) [66] with the optimal (JTT + F + I + G4) model and 1000 bootstrap iterations, *Oryza sativa* were assigned as outgroups. The evolutionary divergent time was calculated using the MCMCTree module in the PAML (v4.9i) software [67] with ‘JC69’ and ‘correlated molecular clock’ model. The analysis of Markov Chain Monte Carlo analysis was performed with the following parameters: ‘burnin 500,000, sampfreq 30, nsample 5,000,000. The reference calibration points were obtained from the TimeTree (http://www.timetree.org/), *S. lycopersicum* vs *S. tuberosum* (5.23–9.4 Mya), *A. thaliana* vs *P. trichocarpa* (98–117 Mya), *O. sativa* vs *S. lycopersicum* (115–308 Mya).

### Whole genome duplication

To discover homologous gene pair, we first performed self-alignment of *R. molle* assembled genome sequence using diamond (v0.9.29.130) (e < 1e-5, C score > 0.5) [63], The C score value was filtered using JCVI software [68]. For intergenomic alignment, we compared *R. molle* genome with *V.vinifera* and four Ericales species (*A. chinensis*, *R. delavayi*, *R. williamsianum*, *R. simsii*). The results were subjected to McscanX (−m 5) to discover syntenic blocks [14]. Macrosynteny between species karyotypes were drawn by JCVI (v0.9.13) [68]. Genome painter images for gene collinearity were drawn using VGSC [69]. The 4DTv (fourfold degenerate synonymous sites of the third codons) values for all the orthologous and paralogous gene pairs were calculated and corrected using HKY substitution model. The synonymous substitution rate (*Ks*) values were calculated using WGD (v1.1.1) [70], and the distribution of the *Ks* values was plotted.

### Identification of TPSs in *R. molle* genome

A TPSs reference database (Additional file 3: Table S23) was established based on the previously characterized terpene synthases which sequences are available in public database. The protein-coding genes database of *R. molle* was screened using tBLASTn with E-value cutoff of 1.0 × 10^− 50^. TPS protein sequences were aligned using MUSLE with default parameters [59], and the neighbor-joining (NJ) tree was constructed using MEGA-X [71] with 1000 replicate bootstrap support. We further analyzed the tandem replication and segmental replication events in genes from TPS family using MCScanX toolkits (Additional file 4: method S2) [14].

### Analysis of the CYP genes from *R. molle*

We downloaded the CYP protein sequences of *A. thaliana* from TAIR (https:// www. Arabidopsis.org/), and used them as queries to search against protein-coding genes database of *R. molle* using BLASTp with the E-value cutoff of 1e^− 40^. The conserved domains of these candidates were further analyzed using Pfam database (http://pfam.xfam.org/). All RmCYP protein sequences were submitted to P450 nomenclature committee (DavidNelson: dnelson@uthsc.edu) to obtain uniform nomenclature. Pseudogenes were identified by finding stop codons or frameshift mutations in their gene sequences, which were assigned by adding a “P” at the end of the original name. For those genes whose identity was over 98%, “a” and “b” were added at the end of their name to distinguish them. The phylogenetic tree of RmCYPs was constructed using FastTree (methods S3) [72]. The gene duplication events of RmCYPs were analyzed by MCScanX toolkits (Additional file 4: methods S2) [14], and the divergence time of each duplicated pair was calculated based on *Ks* value. The heatmap of members from CYP71AU subfamily and TPS candidates were drew by TBtools [73].

### Construction of erg9 haploid deficient mutant yeast strain

To construct deletion cassette, the antibiotic marker gene KanMX flanked by loxP sites was PCR amplified from pUG6 plasmid, then this selectable marker cassette was fused with around 500 bp upstream and downstream homologous regions of the target gene ERG9, which was cloned from the genomic DNA of wild yeast strain INVSc1. The deletion cassette was transformed into diploid wild strain INVSc1 using LiAc method. The yeast transformants were selected on YPD plates supplemented with 300 mg L^− 1^ G418.The stable integration of deletion cassette was verified by PCR amplification. To excise the selectable marker in the deletion cassette, a Cre recombinase expression plastid pSH47 was transformed into mutant strain. Eventually, an ERG9 haploid deficient mutant without selectable marker was obtained, we named it as IN-0.

### Functional characterization diterpene synthase candidates in yeast

To improve GGPP production in yeast cell, we first construct the ERG9 haploid deficient mutant yeast strain IN-0. *BTS1*, *ERG20* and *tHMG1* genes were cloned from the genomic DNA of *S. cerevisiae* strain INVSc1, which was extracted using TIANamp Yeast DNA Kit (TianGen, Beijing, China). *BTS1* and *ERG20* were fused by a GGGS linker and subsequently subcloned into MCS1 of yeast epitope-tagging vector pESC-LEU via In-Fusion cloning to generate construct pESC-LEU-(BTS1-ERG20), and was controlled by GAL10 inducible promoter. The *tHMG1* gene was then subcloned into MCS2 of pESC-LEU-(BTS1-ERG20), and finally we obtained the recombinant plasmid pESC-LEU-(BTS1-ERG20)-tHMG (Additional file 2: Table S20). This plasmid was subsequently transformed into IN-0 to generate yeast strain IN-1using LiAc method [74], which was used as a “chassis” in present study to test enzymatic function of diterpene synthases.

The 5′ and 3′ ends of the *RmTPS1*, *RmTPS2* and *RmTPS5* were cloned using SMARTer RACE 5′/3′ Kit (Clontech) according to the protocol from manufacturer. Total RNA was extracted from leaf by TRIzol reagent, and first-strand cDNA was synthesized by First Strand cDNA synthesis supermix (TransGen Biotech, Beijing, China). The coding sequences of diterpene synthase candidates were cloned from the total cDNA using Q5 high-fidelity DNA polymerase (NEB) and gene-specific primers (Additional file 2: Table S24). The CDS region of these candidates were subcloned into yeast epitope-tagging vector pESC-URA (individually or in combination) by In-Fusion Cloning (Vazyme Biotech, Nanjing, China), they were under the control of the GAL10 or GAL1 inducible promoter, and the inserts were verified by gene sequencing. The generated constructs pESC-URA-RmTPS1, pESC-URA-RmTPS2, pESC-URA-RmTPS1-RmTPS5 and pESC-URA-RmTPS2-RmTPS5 were transformed into yeast strain IN-1 using the LiAc method [74] .Transformants were selected on appropriate selective synthetic dropout (SD) plates and grown at 30 °C for 48 h. The recombinant strains were initially grown in 50 ml SD-His-Trp medium with 2% glucose at 30 °C, 220 rpm, to an OD600 of ~ 0.6–0.8, then were transferred to 100 mL synthetic dropout induction medium with 2% galactose to an initial OD of 0.05. After growing for 72 h at 30 °C, 220 rpm, yeast cell pellets were separated from the medium and extracted with hexane three times. The organic phases were pooled and evaporated using rotary evaporator (Yarong biochemical instrument company, shanghai, China), and finally concentrated to 200 μL in vacuum prior to GC-MS analysis (Additional file 4: method S4). Large-scale shake flask culture was performed to obtain purified product for structure identification by ^1^H NMR, ^13^C NMR (Additional file 4: method S5).

### Genome mining for terpenoid biosynthetic gene clusters

The plantiSMASH web server [75] (default parameters) was used to discover putative biosynthetic gene clusters (BGCs) in *R. molle* genome. The FASTA sequences of assembled genome along with their GFF3 (Generic Feature Format version 3) annotation files were supplied to the web server. Three putative terpenoid biosynthetic gene clusters were discovered through analysis of FASTA+GFF3 format input files.

## Supplementary Information

**Additional file 1: Figure S1.** Estimation of genome size of *R. molle* by *K-mer* analysis. We generated the 21-*mers* frequency distribution of sequencing reads from 350bp insert size library. The x-axis is the depth of 21-mer; the y-axis is the proportion of the frequency at given depth. **Figure S2.** The Hi-C chromatin interaction map for the 13 chromosomes of *R. molle*. LG1-LG13 indicate Lachesis groups 1-13. X and Y axis represent the order positions of scaffolds on corresponding chromosomes**.** The interaction intensity is normalized by the log2 value. The interaction strength is presented by colored bar alongside of the map. **Figure S3.** Functional annotation of the protein-coding genes A. The GO annotation of protein-coding genes. B. The KOG annotation of protein-coding genes. C. The distribution of TFs family in the genome of *R. molle*. **Figure S4.** The proportion of different type LTRs. Gypsy is most abundant, the ratio of Gypsy to Copia is 5.57. **Figure S5.** The insertion time of intact LTRs. In *R. molle* genome, most of the LTR insertion events occurred in the last 2 million years. The species names were abbreviated as follows: *A. chinensis* (*Ath*), *R. delavayi* (*Rde*), *R. molle* (*Rmo*), *R. simsii* (*Rsi*), *R. williamsianum* (*Rwi*). **Figure S6.** Phylogenetic analysis of *R. molle* and ten other related species. The bootstrap value is showed at nodes. **Figure S7.** The syntenic dot plot of the paralogs in *R. molle* genome. This plot showed chromosomal relationship within *R. molle* genome. **Figure S8.** The 4DTv distributions of orthologous genes and paralogous genes in *R. molle* and its related species. Abbreviation: *Rmo-R.molle*; *Ath*-*A. chinensis*; *Rde*- *R. delavayi*; *Rsi*- *R. simsii*; *Rwi*- *R. williamsianum.* **Figure S9.** Proportion of orthologous genes in *R. molle* and ten other plants. **Figure S10.** The GO functional enrichment of *R. molle* unique genes. **Figure S11.** Expanded and contracted gene families among eleven plants. Expanded gene families are showed in green, and contracted gene families are showed in purple. **Figure S12.** The KEGG enrichment of expansion genes in *R. molle* genome. **Figure S13.** The neighbor-joining (NJ) tree of the CPS and KS candidates in *R. molle* genome. PpCPS/KS (*P. patens* copalyl diphosphate synthase /kaurene synthase) was assigned as tree root. The phylogenetic analysis was conducted by MEGA-X using the poisson model and 1000 bootstrap replicates. Bootstrap values ≥ 70% are indicated with lilac circle, the alignment of N-terminal and C-terminal conserved sequences were displayed alongside the tree. **Figure S14.** Constrution of ERG9 deletion cassette and primers design strategy. The upstream and downstream homologous region of *ERG9* were amplified from the genome of yeast strain INVSc1, using primers (C,D) and primers (E,F), respectively. Primer D and E harboured the upstream and downstream sequences of pUG6 loxP region, respectively. The homologous regions were fused with the loxP-*kanMX*-loxP selectable marker cassette, which was cloned from pUG6 plasmid. Primers M-A and M-B were located within *kanMX* marker, which were used to screen the positive. transformants. All the above-mentioned primers were listed in table 30. **Figure S15.** The mass spectra of three products yielded by RmTPS1 and RmTPS5. **Figure S16.** 1H NMR spectrum of ent-copalol recorded in pyridine-d5 (C5D5N) at 25 °C. **Figure S17.** 13C NMR spectrum of ent-copalol recorded in pyridine-d5 (C5D5N) at 25 °C. **Figure S18.** 1H NMR spectrum of 16α-hydroxy-ent-kaurene recorded in pyridine-d5 (C5D5N) at 25 °C. 12. **Figure S18** 1H NMR spectrum of 16α-hydroxy-ent-kaurene recorded in pyridine-d5 (C5D5N) at 25 °C. **Figure S19.** 13C NMR spectrum of 16α-hydroxy-ent-kaurene recorded in pyridine-d5 (C5D5N) at 25 °C. **Figure S20.** The structure of grayanoid compounds isolated from *R. molle*. These compounds have an α-hydroxy group at the C-16. **Figure S21**. Phylogenetic relationships and conserved motifs feature of RmCYPs. **Figure S22.** The scatter plot of Ka/Ks ratio of homologous CYP gene pairs. Blue dots indicate tandem duplicated pairs and yellow dots indicate segmental duplicated pairs. **Figure S23.** The gene structure feature and conserved motifs of members from CYP71AU subfamily. 0, 1 and 2 indicate the types of intron phase. The conserved motifs of each CYP71AUs were also displayed.

**Additional file 2: Table S1.** The statistic results of survey for *R. molle* genome. **Table S2.** Sequencing data statistic results of *R. molle* genome. **Table S3.** The length distribution of subreads. **Table S4.** The statistical results of Hi-C assembly. **Table S5.** Assessment of coverage by mapping reads from Illumina sequencing. **Table S6.** BUSCO assessment of the genome assembly. **Table S7.** CEGMA assessment of the genome assembly. **Table S8.** Summary of predicted protein-coding genes in the *R. molle* genome. **Table S9.** Summary of gene structure prediction of *R. molle.* **Table S10.** Functional annotation of the protein-coding genes. **Table S11.** Non-coding RNA in *R. molle* genome. **Table S12.** Summary of repetitive elements in *R. molle* genome. **Table S13.** Statistic results of synteny within *R. molle* genome. **Table S14.** Summary of the gene families among *R. molle* and ten other plant species. **Table S17.** The summary of the TPS family in *R. molle* genome. **Table S18.** The duplicated pairs of terpene synthases in *R. molle* genome. **Table S19.** The distribution of TPS tandem clusters in chromosomes. **Table S20.** All plasmids and strains used in this study. **Table S24.** All primers used in this study.

**Additional file 3: Table S15.** The GO enrichment analysis of expanded genes in *R. molle.* **Table S16.** The KEGG enrichment analysis of expanded genes in *R. molle.* **Table S21.** The family classification of RmCYP genes. **Table S22.** The duplicated pairs of cytochrome P450 in *R. molle* genome. **Table S23.** Terpene synthase reference database used in the current study.

**Additional file 4. Methods S1.** RNA sequencing and assembly. **Methods S2.** Gene duplication events and selective pressure analyses. **Methods S3.** Construction of CYPs phylogenetic tree and analysis of gene structure. **Methods S4.** Gas chromatographic-mass spectrometric analysis. **Methods S5.** Product purification and structure identification.

## Data Availability

The data that support the findings of this study have been deposited into CNGB Sequence Archive (CNSA) of China National GeneBank DataBase (CNGBdb) with project accession ID: CNP0001888. The accession numbers of each sequencing are as follow: PacBio sequencing reads (CNR0386171, CNR0386172, CNR0386173, CNR0386174); Illumina HiSeq sequencing reads (CNR0386175); Hi-C sequencing reads (CNR0386178, CNR038617,); Transcriptome sequencing reads (CNR0386176, CNR0386177); Genome assembly (CNA0022751). All the raw sequencing data have also been deposited under NCBI BioProject number PRJNA664478. The *R. molle* whole genome shotgun project has been deposited at GenBank under the accession number JACYLG000000000.
