# Supplementary material for: Chromosome-scale genome assembly of Rhododendron molle provides insights into its evolution and terpenoid biosynthesis
Source: BMC Plant Biol. 2022 Jul 15;22:342. doi: 10.1186/s12870-022-03720-8 (PMC9284817; doi:10.1186/s12870-022-03720-8)
Supplement: Supplementary file 1 — Additional file 1: Figure S1. Estimation of genome size of R. molle by K-mer analysis. We generated the 21-mers frequency distribution of sequencing reads from 350bp insert size library. The x-axis is the depth of 21-mer; the y-axis is the proportion of the frequency at given depth. Figure S2. The Hi-C chromatin interaction map for the 13 chromosomes of R. molle. LG1-LG13 indicate Lachesis groups 1-13. X and Y axis represent the order positions of scaffolds on corresponding chromosomes. The interaction intensity is normalized by the log2 value. The interaction strength is presented by colored bar alongside of the map. Figure S3. Functional annotation of the protein-coding genes A. The GO annotation of protein-coding genes. B. The KOG annotation of protein-coding genes. C. The distribution of TFs family in the genome of R. molle. Figure S4. The proportion of different type LTRs. Gypsy is most abundant, the ratio of Gypsy to Copia is 5.57. Figure S5. The insertion time of intact LTRs. In R. molle genome, most of the LTR insertion events occurred in the last 2 million years. The species names were abbreviated as follows: A. chinensis (Ath), R. delavayi (Rde), R. molle (Rmo), R. simsii (Rsi), R. williamsianum (Rwi). Figure S6. Phylogenetic analysis of R. molle and ten other related species. The bootstrap value is showed at nodes. Figure S7. The syntenic dot plot of the paralogs in R. molle genome. This plot showed chromosomal relationship within R. molle genome. Figure S8. The 4DTv distributions of orthologous genes and paralogous genes in R. molle and its related species. Abbreviation: Rmo-R.molle; Ath-A. chinensis; Rde- R. delavayi; Rsi- R. simsii; Rwi- R. williamsianum. Figure S9. Proportion of orthologous genes in R. molle and ten other plants. Figure S10. The GO functional enrichment of R. molle unique genes. Figure S11. Expanded and contracted gene families among eleven plants. Expanded gene families are showed in green, and contracted gene families are showed in [file 12870_2022_3720_MOESM1_ESM.pdf]

# Chromosome-scale genome assembly of *Rhododendron molle* provides insight into its evolution and terpenoids biosynthesis

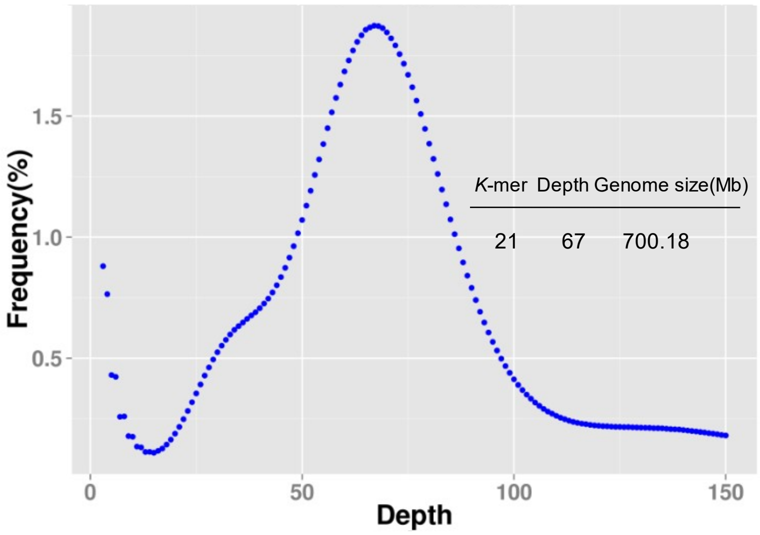


**Figure S1.** Estimation of genome size of *R. molle* by *K-mer* analysis. We generated the 21-*mers* frequency distribution of sequencing reads from 350bp insert size library. The x-axis is the depth of 21-mer; the y-axis is the proportion of the frequency at given depth

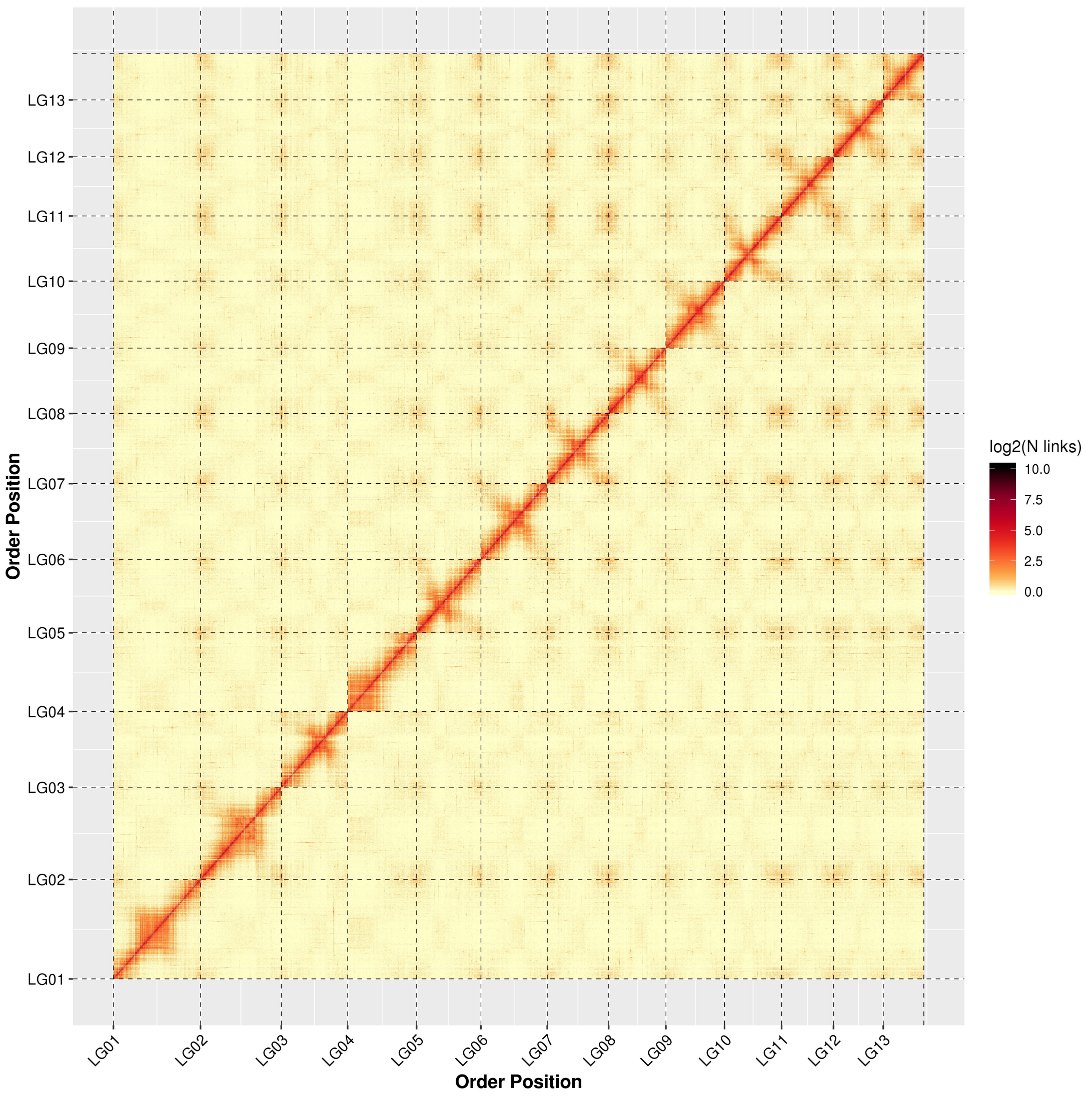


**Figure S2.** The Hi-C chromatin interaction map for the 13 chromosomes of *R. molle*. LG1-LG13 indicate Lachesis groups 1-13. X and Y axis represent the order positions of scaffolds on corresponding chromosomes**.** The interaction intensity is normalized by the log2 value. The interaction strength is presented by colored bar alongside of the map.

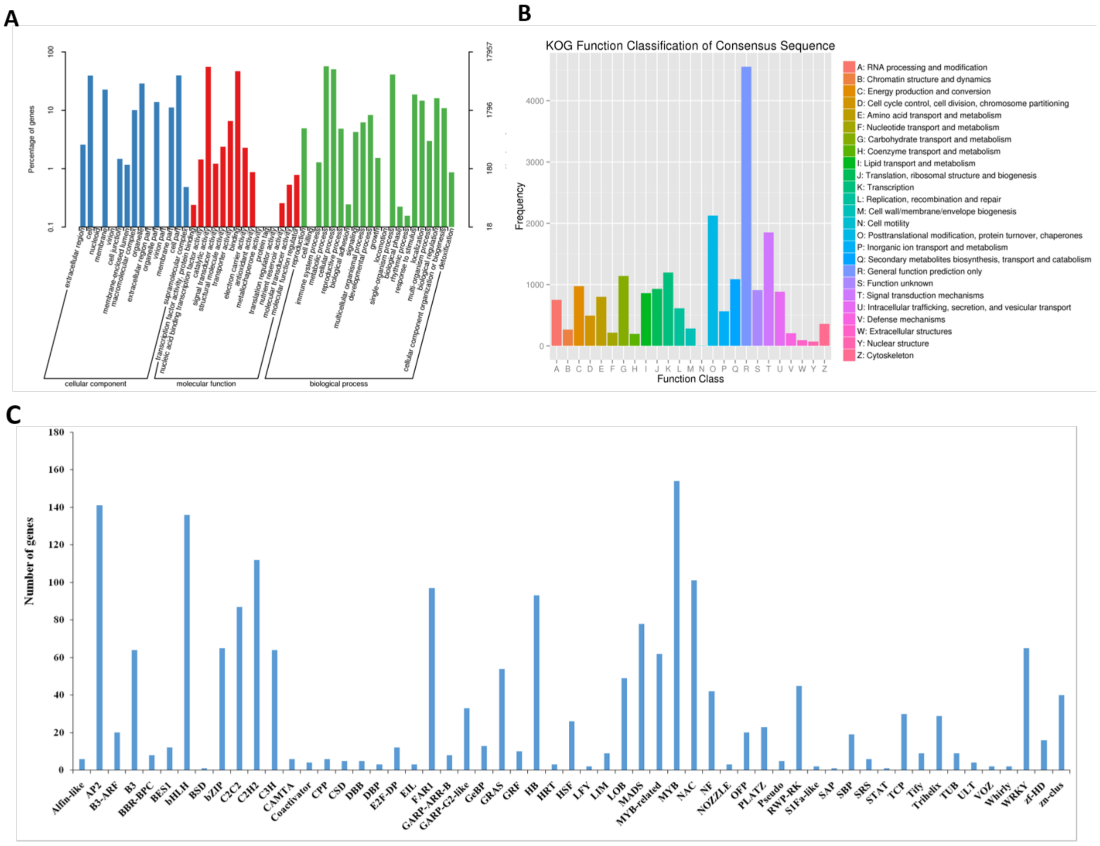


**Figure S3.** Functional annotation of the protein-coding genes A. The GO annotation of protein-coding genes. B. The KOG annotation of protein-coding genes. C. The distribution of TFs family in the genome of *R. molle*.

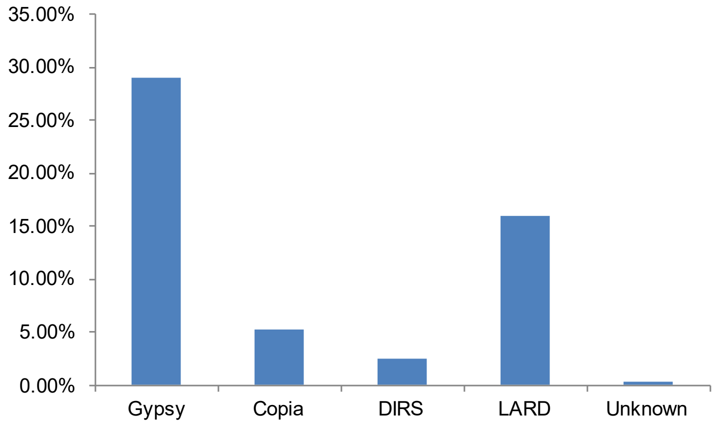


**Figure S4.** The proportion of different type LTRs. Gypsy is most abundant, the ratio of Gypsy to Copia is 5.57.

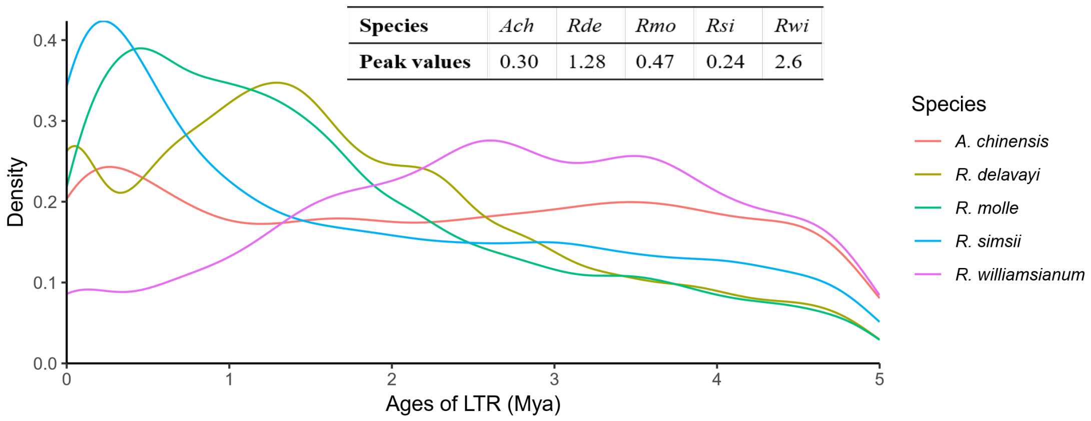


**Figure S5.** The insertion time of intact LTRs. In *R. molle* genome, most of the LTR insertion events occurred in the last 2 million years. The species names were abbreviated as follows: *A. chinensis* (*Ath*), *R. delavayi* (*Rde*), *R. molle* (*Rmo*), *R. simsii* (*Rsi*), *R. williamsianum* (*Rwi*).

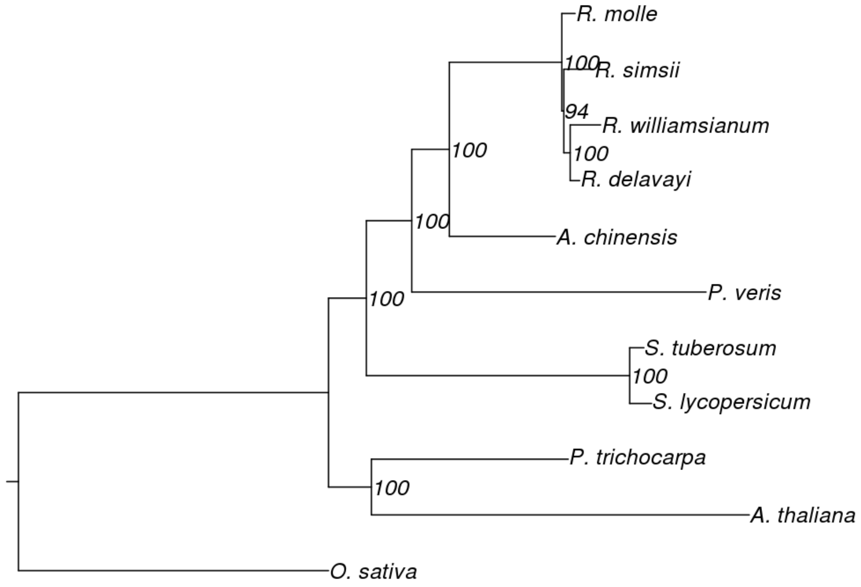


**Figure S6.** Phylogenetic analysis of *R. molle* and ten other related species. The bootstrap value is showed at nodes

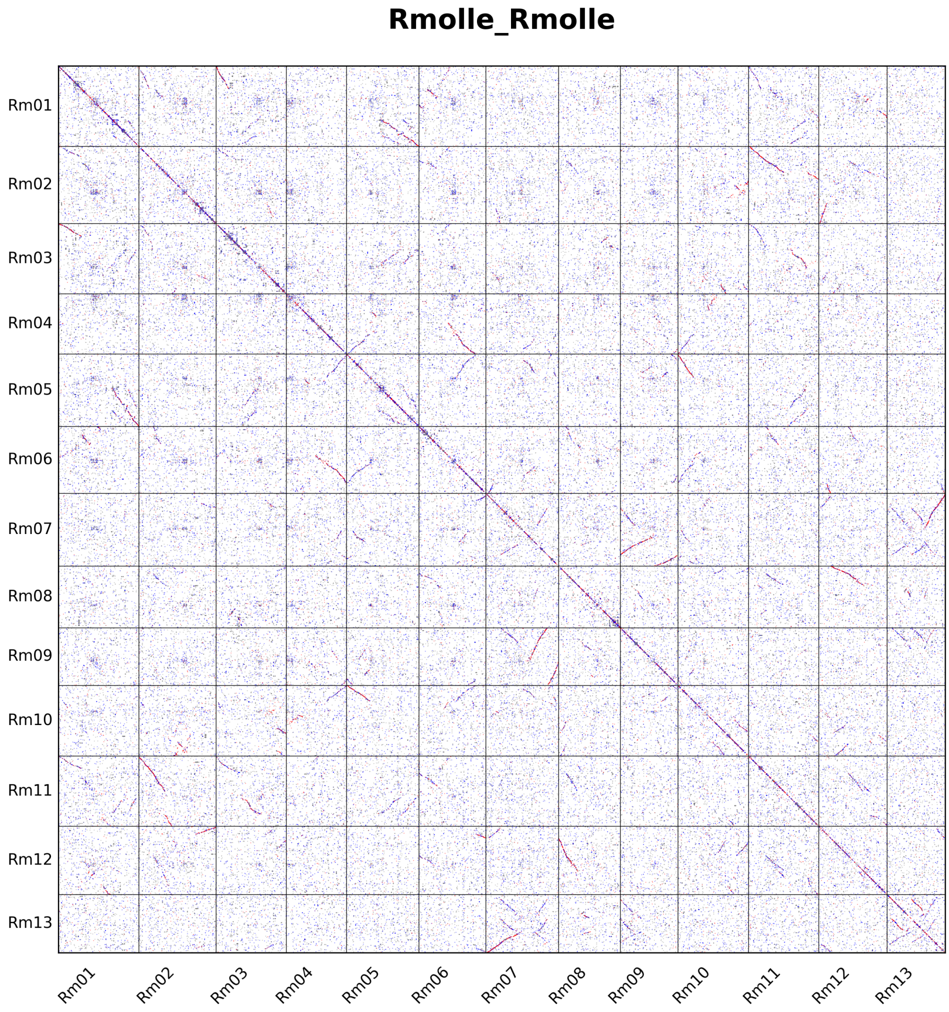


**Figure S7.** The syntenic dot plot of the paralogs in *R. molle* genome. This plot showed chromosomal relationship within *R. molle* genome.

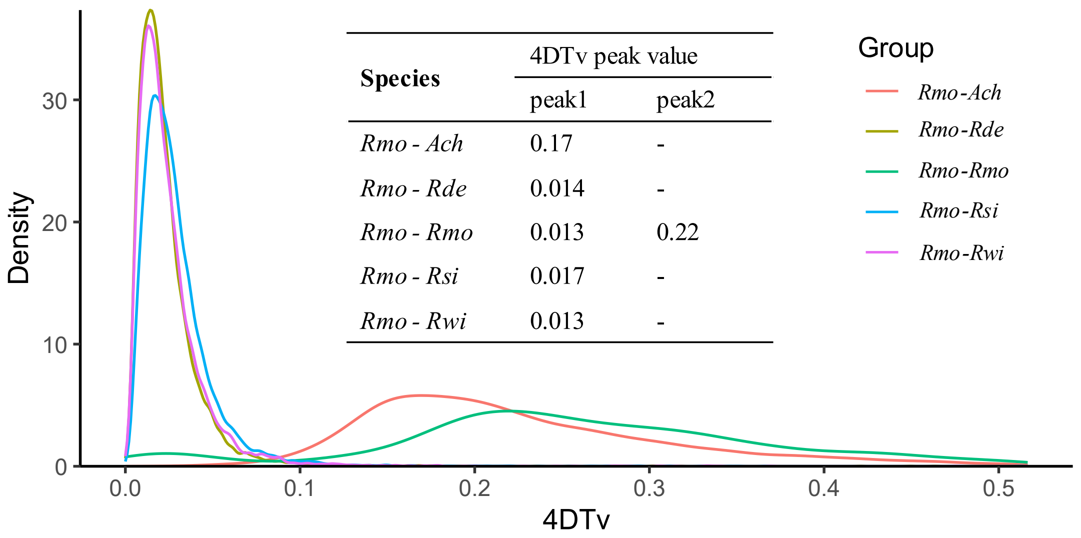


**Figure S8. The 4DTv distributions of orthologous genes and paralogous genes in R. molle and its related species.** Abbreviation: *Rmo-R.molle*; *Ath-A. chinensis*; *Rde- R. delavayi*; *Rsi- R. simsii*; *Rwi- R. williamsianum*

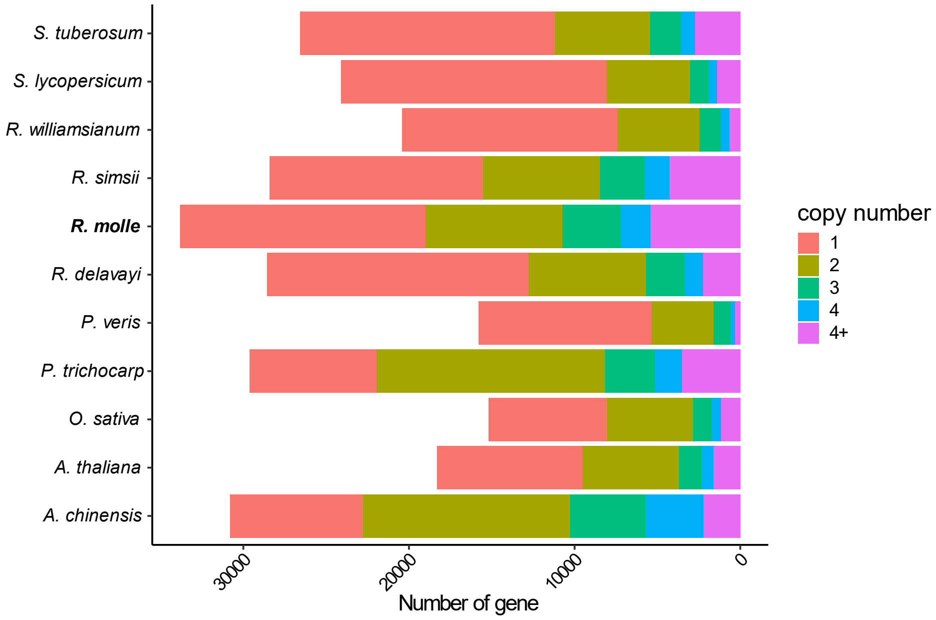


**Figure S9.** Proportion of orthologous genes in *R. molle* and ten other plants

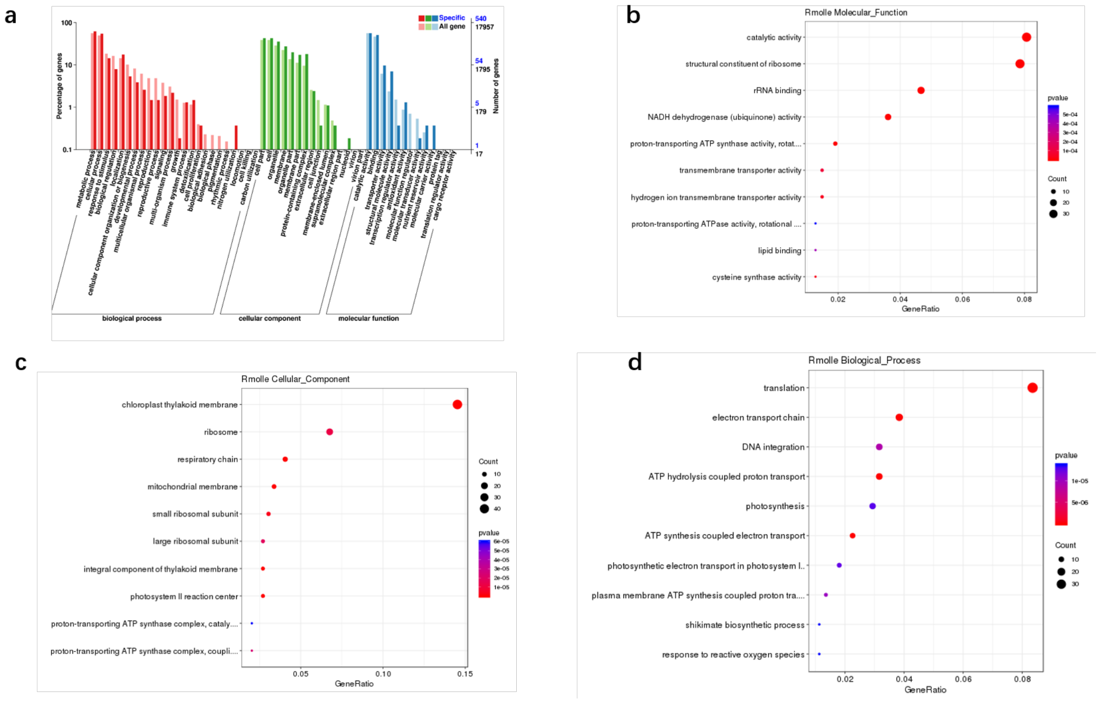


**Figure S10.** The GO functional enrichment of *R. molle* unique genes.

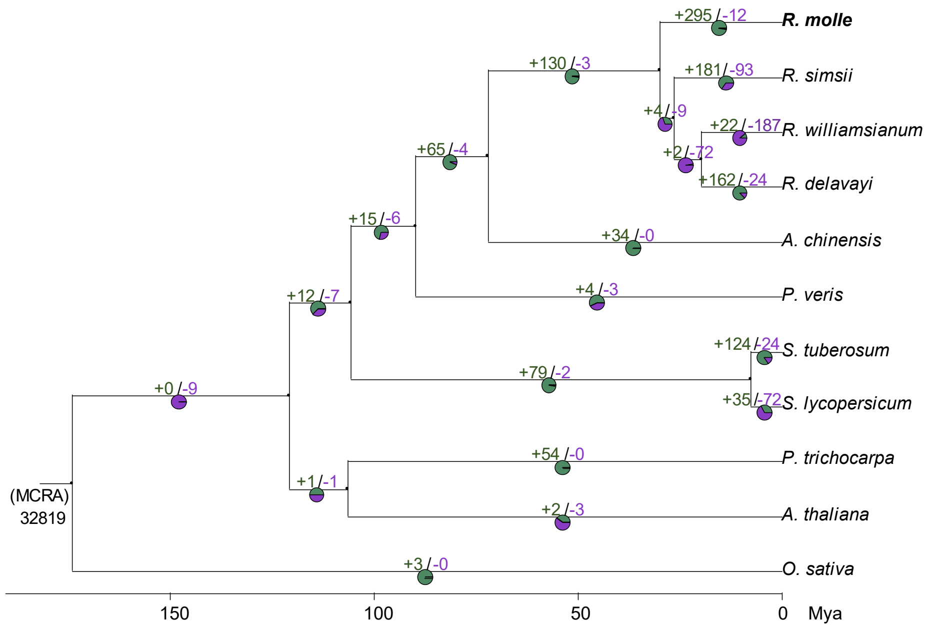


**Figure S11.** Expanded and contracted gene families among eleven plants. Expanded gene families are showed in green, and contracted gene families are showed in purple.

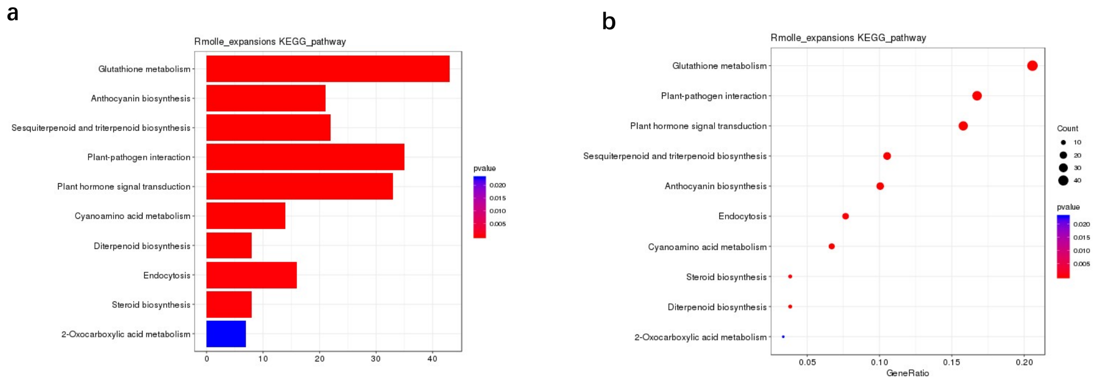


**Figure S12.** The KEGG enrichment of expansion genes in *R. molle* genome

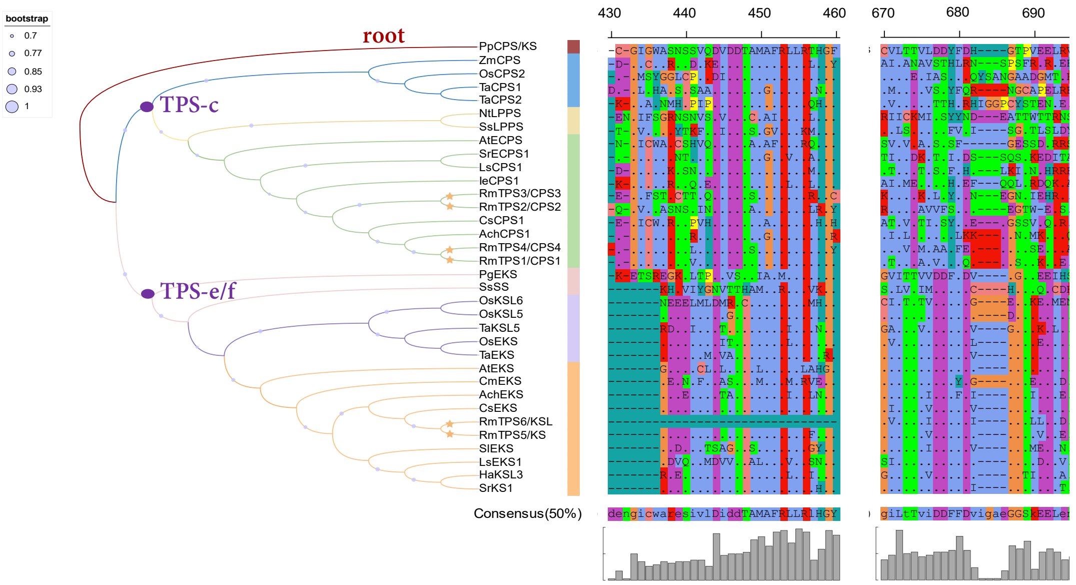


**Figure S13.** The neighbor-joining (NJ) tree of the CPS and KS candidates in *R. molle* genome. PpCPS/KS (*P. patens* copalyl diphosphate synthase /kaurene synthase) was assigned as tree root. The phylogenetic analysis was conducted by MEGA-X using the poisson model and 1000 bootstrap replicates. Bootstrap values ≥ 70% are indicated with lilac circle, the alignment of N-terminal and C-terminal conserved sequences were displayed alongside the tree.

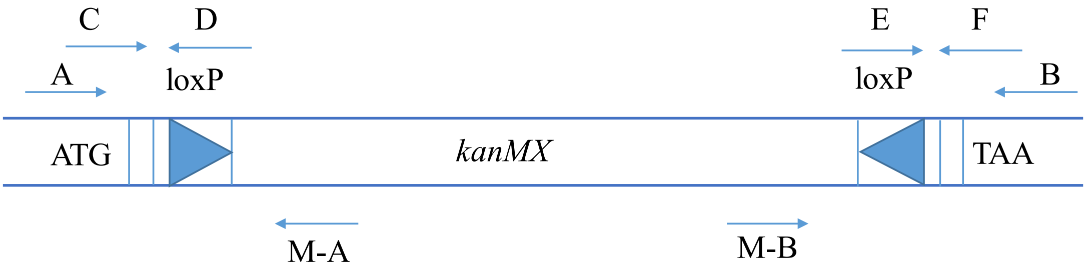


**Figure S14.** Constrution of ERG9 deletion cassette and primers design strategy. The upstream and downstream homologous region of *ERG9* were amplified from the genome of yeast strain INVSc1, using primers (C,D) and primers (E,F), respectively. Primer D and E harboured the upstream and downstream sequences of pUG6 loxP region, respectively.The homologous regions were fused with the loxP-*kanMX*-loxP selectable marker cassette, which was cloned from pUG6 plasmid. Primers M-A and M-B were located within *kanMX* marker, which were used to screen the positive transformants. All the above-mentioned primers were listed in table 30

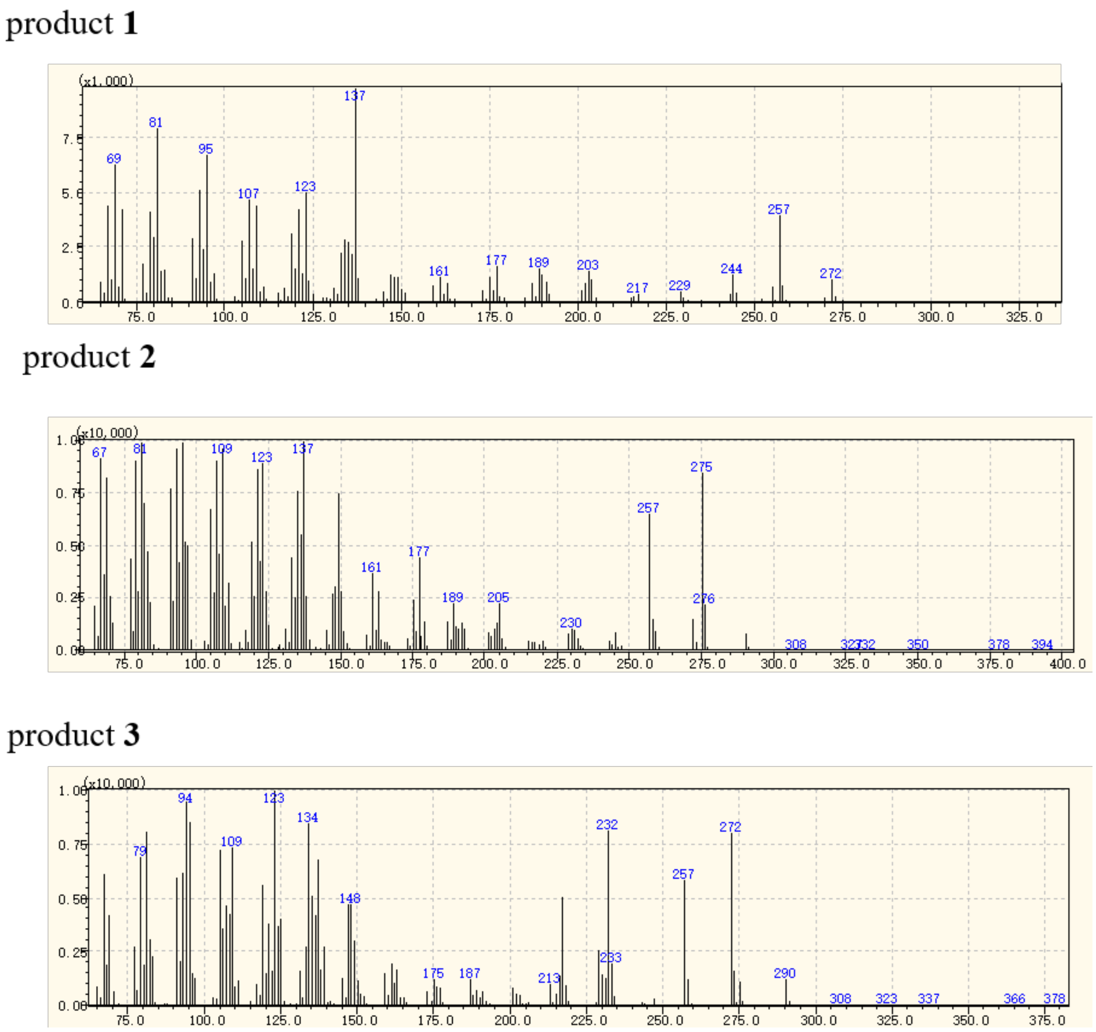


**Figure S15.** The mass spectra of three products yielded by RmTPS1 and RmTPS5

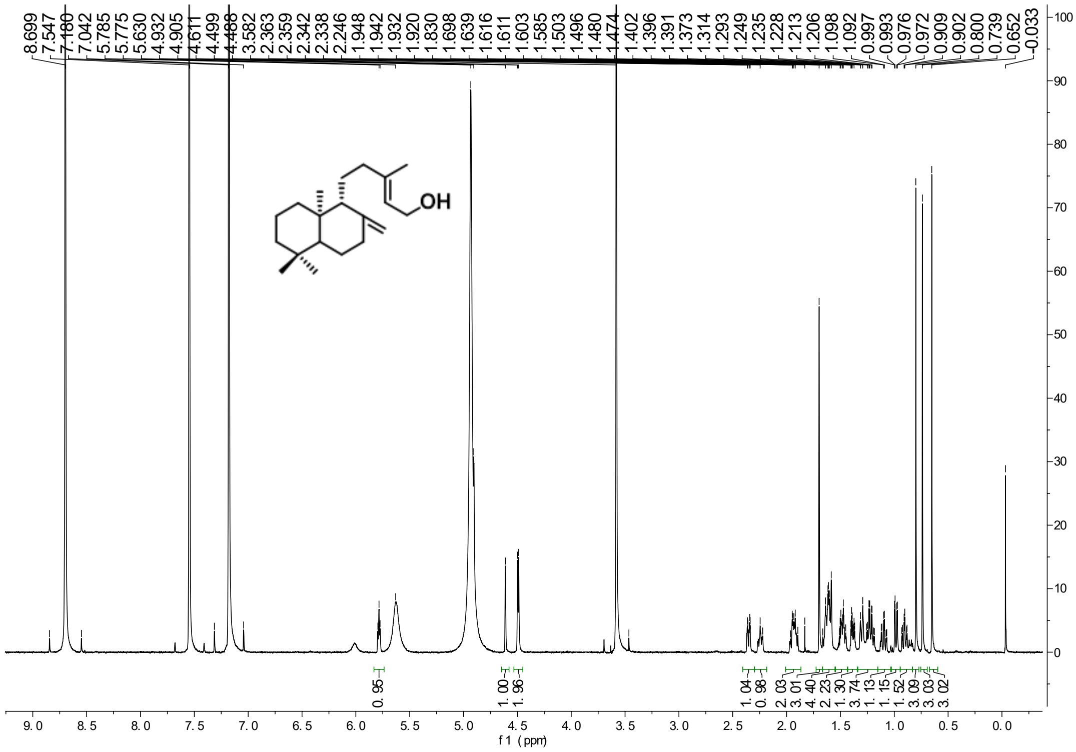


**Figure S16.** $^{1}$H NMR spectrum of ent-copalol recorded in pyridine-d5 (C5D5N) at 25 °C

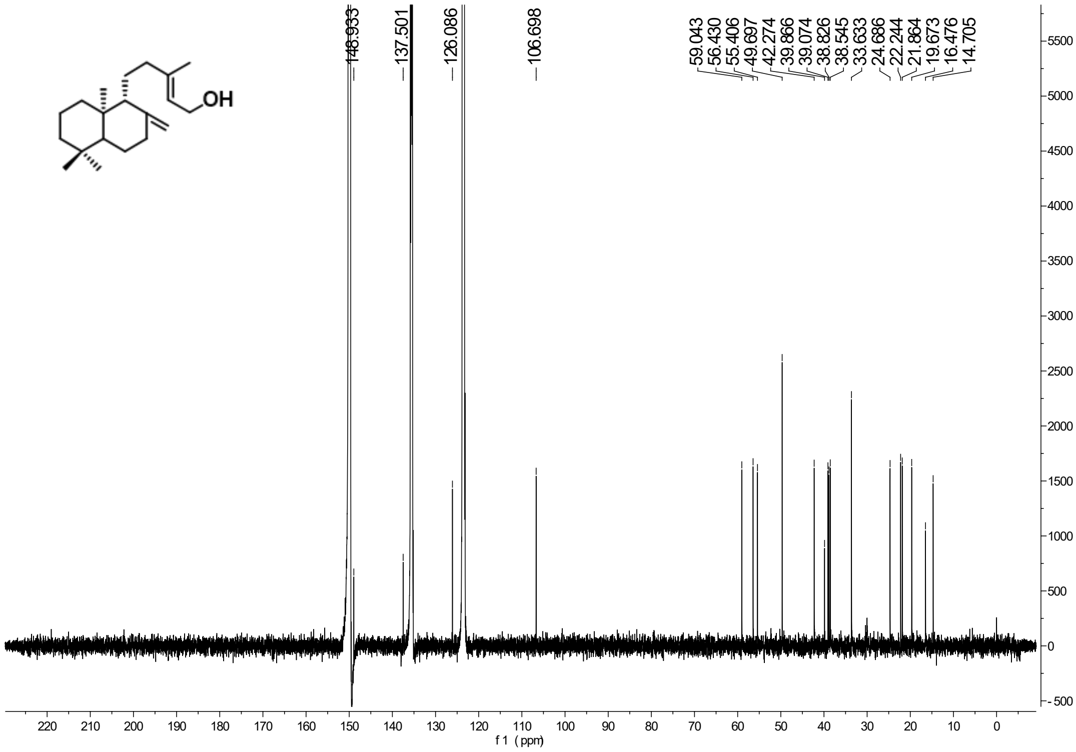


**Figure S17.** $^{13}$C NMR spectrum of ent-copalol recorded in pyridine-d5 (C5D5N) at 25 °C

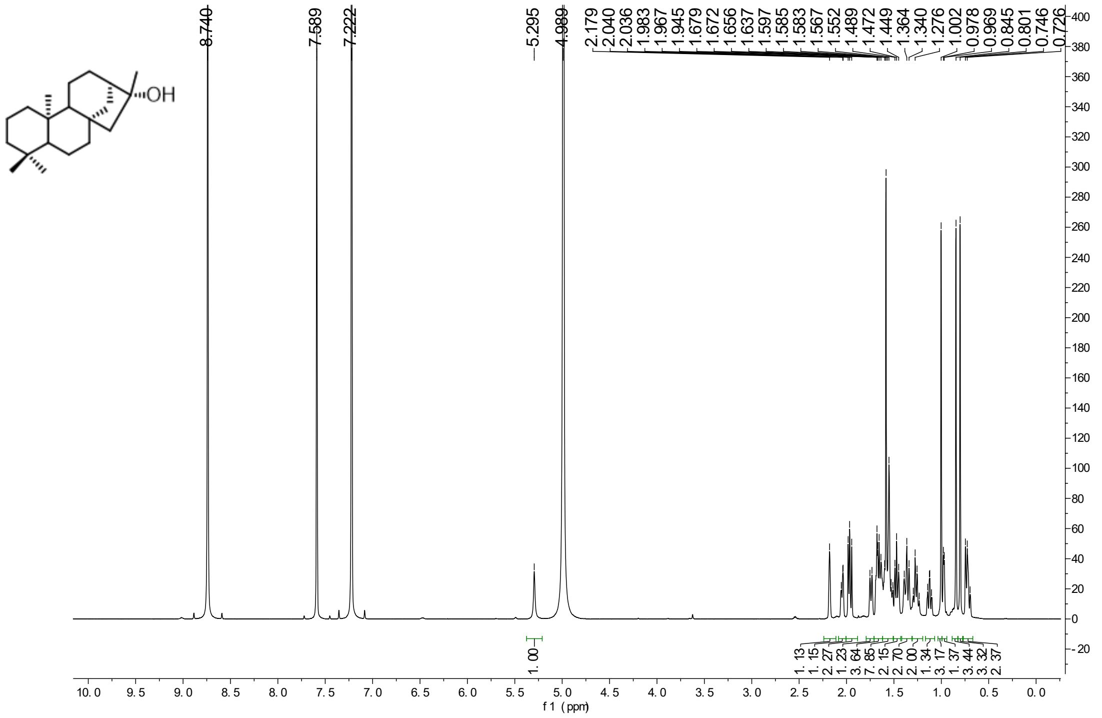


**Figure S18** $^{1}$H NMR spectrum of 16α-hydroxy-ent-kaurene recorded in pyridine-d5 (C5D5N) at 25 °C

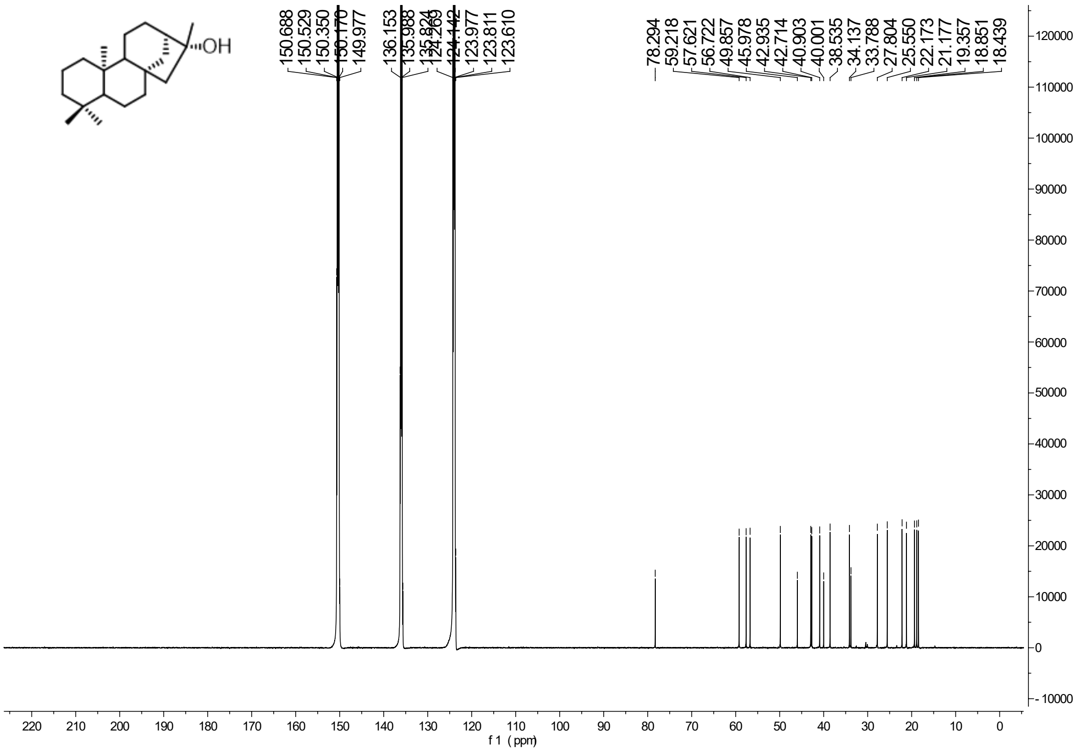


**Figure S19.** $^{13}$C NMR spectrum of 16α-hydroxy-ent-kaurene recorded in pyridine-d5 (C5D5N) at 25 °C

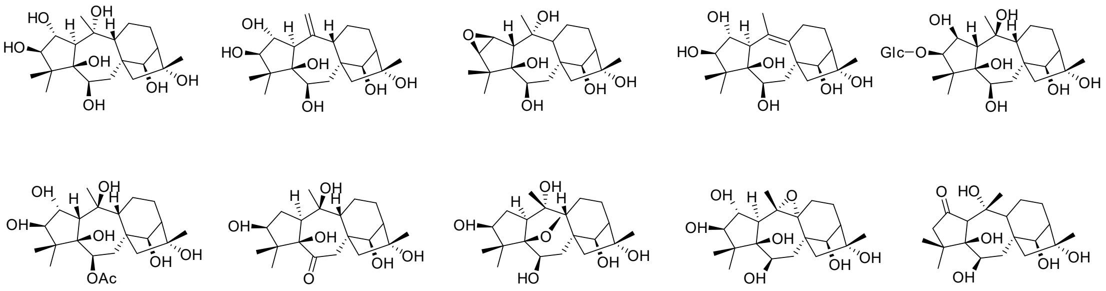


**Figure S20.** The structure of grayanoid compounds isolated from *R. molle*. These compounds have an α-hydroxy group at the C-16.

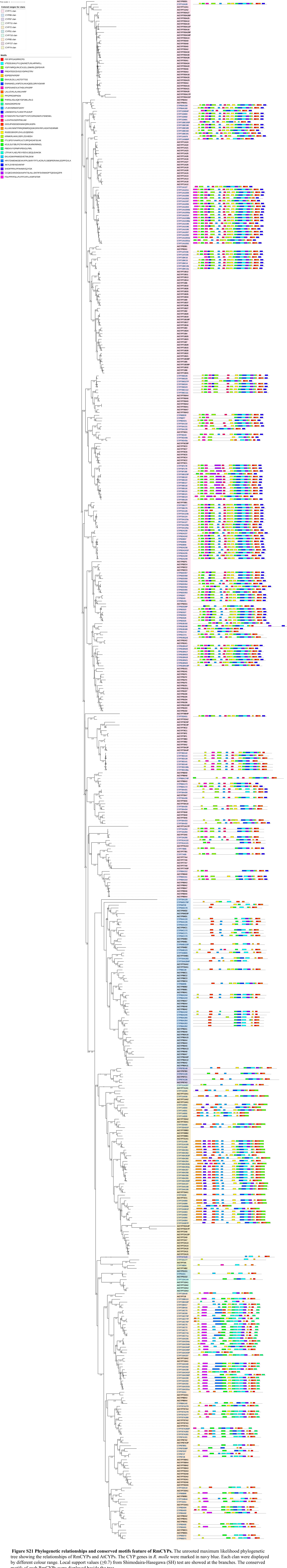

**Figure S21 Phylogenetic relationships and conserved motifs feature of RmCYPs.** The unrooted maximum likelihood phylogenetic tree showing the relationships of RmCYPs and AtCYPs. The CYP genes in *R. molle* were marked in navy blue. Each clan were displayed by different colour range. Local support values (≥0.7) from Shimodaira-Hasegawa (SH) test are showed at the branches. The conserved motifs of each RmCYPs were displayed beside the tree.

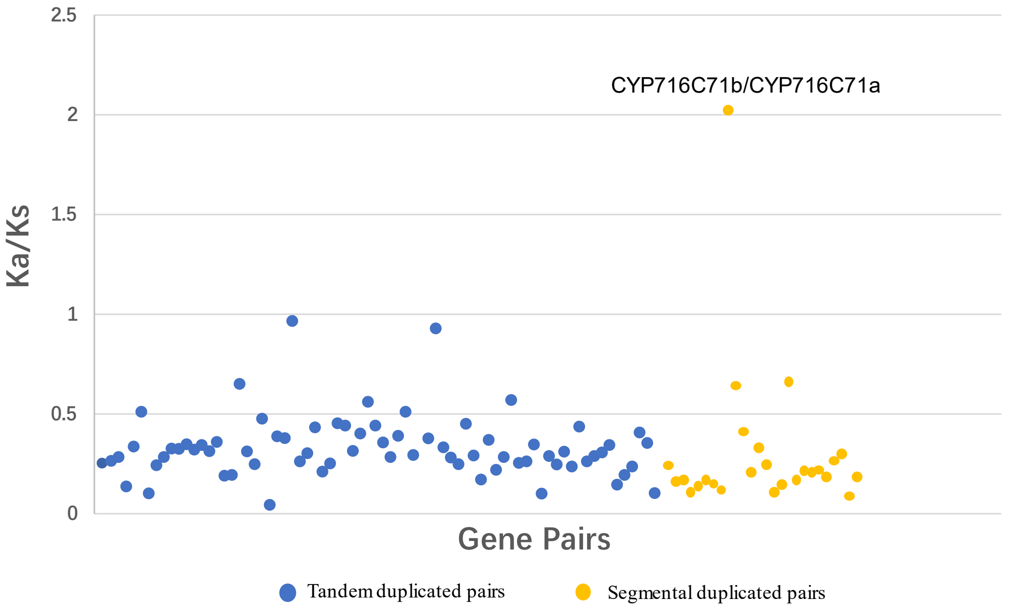


**Figure S22.** The scatter plot of Ka/Ks ratio of homologous CYP gene pairs. Blue dots indicate tandem duplicated pairs and yellow dots indicate segmental duplicated pairs.

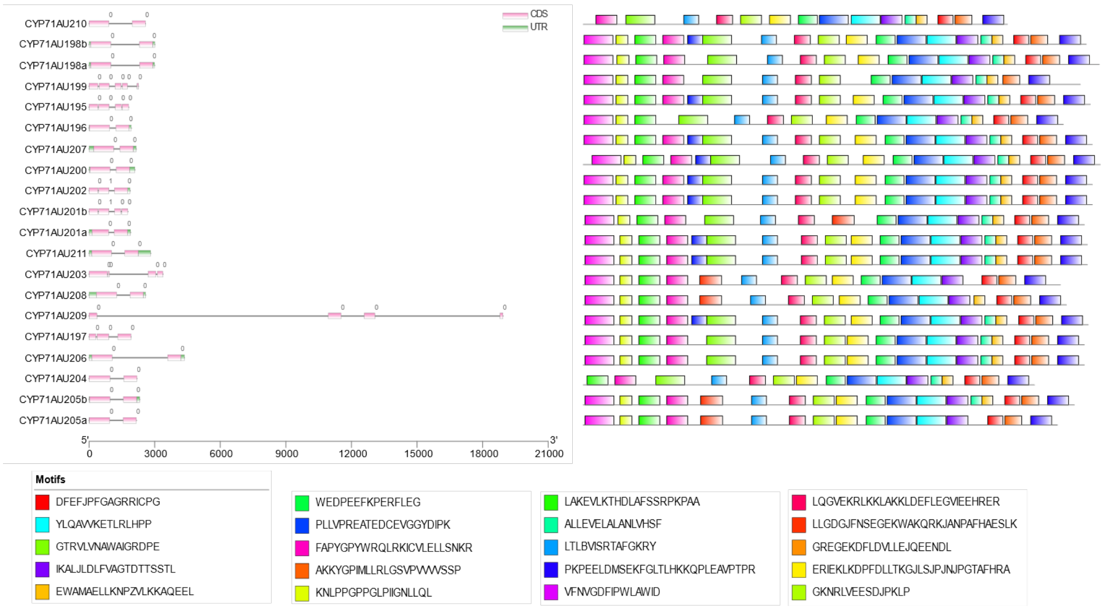


**Figure S23.** The gene structure feature and conserved motifs of members from CYP71AU subfamily. 0, 1 and 2 indicate the types of intron phase. The conserved motifs of each CYP71AUs were also displayed.
