# Supplementary material for: Chromosome-scale genome assembly of Rhododendron molle provides insights into its evolution and terpenoid biosynthesis
Source: BMC Plant Biol. 2022 Jul 15;22:342. doi: 10.1186/s12870-022-03720-8 (PMC9284817; doi:10.1186/s12870-022-03720-8)
Supplement: Supplementary file 2 — Additional file 2: Table S1. The statistic results of survey for R. molle genome. Table S2. Sequencing data statistic results of R. molle genome. Table S3. The length distribution of subreads. Table S4. The statistical results of Hi-C assembly. Table S5. Assessment of coverage by mapping reads from Illumina sequencing. Table S6. BUSCO assessment of the genome assembly. Table S7. CEGMA assessment of the genome assembly. Table S8. Summary of predicted protein-coding genes in the R. molle genome. Table S9. Summary of gene structure prediction of R. molle. Table S10. Functional annotation of the protein-coding genes. Table S11. Non-coding RNA in R. molle genome. Table S12. Summary of repetitive elements in R. molle genome. Table S13. Statistic results of synteny within R. molle genome. Table S14. Summary of the gene families among R. molle and ten other plant species. Table S17. The summary of the TPS family in R. molle genome. Table S18. The duplicated pairs of terpene synthases in R. molle genome. Table S19. The distribution of TPS tandem clusters in chromosomes. Table S20. All plasmids and strains used in this study. Table S24. All primers used in this study. [file 12870_2022_3720_MOESM2_ESM.docx]

**Table S1.** The statistic results of survey for *R. molle* genome

| **Species** | **Total**  **base**  **(Gb)** | ***K-***  ***mer*** | ***K-***  ***mer***  **depth** | ***K-mer* number** | **Genome**  **Size (Mb)** | **Heterozygous**  **ratio (%)** | **Repeat**  **Ratio**  **(%)** | **GC content (%)** |
| --- | --- | --- | --- | --- | --- | --- | --- | --- |
| *R. molle* | 58.43 | 21 | 67 | 47,474,641,778 | 700.18 | 0.44 | 53.17 | 40.89 |

**Table S2.** Sequencing data statistic results of *R. molle* genome

| **Pair-end libraties** | **Total data (Gb)** | **%≥Q30** | **Insert size** | **Sequence coverage (×)** |
| --- | --- | --- | --- | --- |
| Illumina | 58.43 | 93.62 | 350bp | 83.45 |
| Pacbio | 49.48 | 93.21 | 20 kb | 66.48 |
| Hi-C | 50 | 93.28 |  | 67 |

**Table S3.** The length distribution of subreads

| **Length (bp)** | **Number** | **Total length (bp)** | **Average length (bp)** |
| --- | --- | --- | --- |
| 500~2,000 | 336,933 | 416,140,098 | 1,235.08 |
| 2000~4,000 | 399,342 | 1,187,980,286 | 2,974.84 |
| 4000~6,000 | 335,915 | 1,669,649,185 | 4,970.45 |
| 6000~8,000 | 288,671 | 2,013,677,442 | 6,975.68 |
| 8000~10,000 | 252,503 | 2,267,715,128 | 8,980.94 |
| 10000~12,000 | 235,165 | 2,586,076,811 | 10,996.86 |
| 12000~14,000 | 228,666 | 2,970,551,100 | 12,990.79 |
| 14000~16,000 | 209,260 | 3,134,546,571 | 14,979.20 |
| 16000~18,000 | 184,143 | 3,126,054,971 | 16,976.24 |
| 18000~ | 1,061,318 | 30,112,294,749 | 28,372.55 |
| **Total** | **3,531,916** | **49,484,686,341** |  |

**Table S4.** The statistical results of Hi-C assembly

| **Group** | **Sequence Number** | **Sequence Length (bp)** |
| --- | --- | --- |
| LG01 | 315 | 76,722,183 |
| LG02 | 218 | 71,938,779 |
| LG03 | 209 | 59,218,889 |
| LG04 | 219 | 59,451,972 |
| LG05 | 198 | 57,682,500 |
| LG06 | 178 | 56,407,428 |
| LG07 | 132 | 51,768,582 |
| LG08 | 183 | 51,134,999 |
| LG09 | 172 | 50,824,541 |
| LG10 | 131 | 48,198,790 |
| LG11 | 98 | 43,428,117 |
| LG12 | 106 | 42,138,258 |
| LG13 | 92 | 35,034,415 |
| Total Clustered Sequences | 2,251 | 703,949,453 |
| Total Oriented Sequences | 1,240 | 620,889,771 |

**Table S5.** Assessment of coverage by mapping reads from Illumina sequencing

| **Total reads** | **Mapped reads** | **Mapped (%)** | **Properly mapped reads** | **Properly mapped (%)** |
| --- | --- | --- | --- | --- |
| 390,324,354 | 385,392,994 | 98.74 | 356,610,264 | 91.36 |

Total Reads: The number of Clean Reads; Mapped reads: The reads mapped into assembled genome; Properly mapped reads: Both the paired-end reads mapped into assembled genome and the distance was consistent with the length distribution of the sequencing fragments

**Table S6.** BUSCO assessment of the genome assembly

| **Species** | **BUSCO assessment results** |
| --- | --- |
| *R. molle* | C:1309 (90.90%), S: 1172(81.39%); D:137(9.51%), F:27(1.88%), M:104(7.22%), T:1440 |

C：Complete BUSCOs; S: Complete and single-copy BUSCOs; D：Complete and duplicated BUSCOs; F：Fragmented BUSCOs; M：Missing BUSCOs; T: Total lineage BUSCOs

**Table S7.** CEGMA assessment of the genome assembly

| **Species** | **Number of 458 CEG present in assembly** | **% Completeness** | **Number of 248 highly conserved CEGs present** | **% Completeness** |
| --- | --- | --- | --- | --- |
| *R. molle* | 449 | 98.03% | 237 | 95.56% |

**Table S8.** Summary of predicted protein-coding genes in the *R. molle* genome

| **Gene set** | **Software** | **Species** | **Gene number** |
| --- | --- | --- | --- |
| De novo | Genscan |  | 68,705 |
|  | Augustus |  | 80,446 |
|  | GlimmerHMM |  | 72,238 |
|  | GeneID |  | 68,705 |
|  | SNAP |  | 74,083 |
|  |  | *Arabidopsis thaliana* | 27,765 |
| Homology-based | GeMoMa | *Actinidia chinensis* | 29,046 |
|  |  | *Rhododendron delavayi* | 47,398 |
|  | PASA |  | 55,200 |
| RNAseq | GeneMarkS-T |  | 40,636 |
|  | TransDecoder |  | 75,402 |
| **Integration** | **EVM** |  | **39,288** |

**Table S9.** Summary of gene structure prediction of *R. molle*

| **Species** | **Number** | **GeneLen**  **(bp)** | **AveGeneLen (bp)** | **CDSLen**  **(bp)** | **AveCDS**  **Len (bp)** | **IntronLen (bp)** | **AveIntron**  **Len (bp)** |
| --- | --- | --- | --- | --- | --- | --- | --- |
| *R. molle* | 39,288 | 186,725,348 | 4,752.73 | 47,687,013 | 1,213.78 | 123,635,209 | 3,146.89 |

GeneLen: gene length; AveGeneLen: average length of gene; CDSLen：CDS length；AveCDSLen：average length of CDS；IntronLen (bp)：Intron length; AveIntronLen(bp): average length of intron

**Table S10.** Functional annotation of the protein-coding genes

| **database** | **Annotated number** | **Annotated Percent (%)** |
| --- | --- | --- |
| Nr | 34,659 | 88.22% |
| GO | 17,957 | 45.71% |
| KOG | 18,979 | 48.31% |
| TrEMBL | 33,433 | 85.10% |
| KEGG | 11,492 | 29.25% |
| **All annotated** | **34,743** | **88.43%** |

**Table S11.** Non-coding RNA in *R. molle* genome

| **Type** | **Numbers** | **Family**  **numbers** | **Total**  **length(bp)** | **Average**  **length (bp)** |  |  |
| --- | --- | --- | --- | --- | --- | --- |
| miRNA | 103 | 21 | 11,597 | 112.59 |  |  |
| tRNA | 667 | 25 | 918,581 | 4592.9 |  |  |
| rRNA | 288 | 4 | 240,451 | 834.89 |  |  |
| snRNA | 230 | 8 | 34,544 | 150.19 |  |  |
| snoRNA | 1,340 | 2 | 144,082 | 107.52 |  |  |
|  | | |  | | |  |

**Table S12.** Summary of repetitive elements in *R. molle* genome

| **Type** | | **Number** | **Length**（**bp**） | **Percentage (%)** |
| --- | --- | --- | --- | --- |
| **Class I: Retroelement** |  | 875,824 | 395,724,732 | 53.15 |
| LTR Retrotransposon | DIRS | 27,830 | 18,369,661 | 2.47 |
|  | LARD | 434,467 | 119,496,149 | 16.05 |
|  | LTR/Copia | 83,587 | 38,770,357 | 5.21 |
|  | LTR/ Gypsy | 212,175 | 216,276,250 | 29.05 |
|  | LTR/Unknown | 4,913 | 2,768,707 | 0.37 |
| non-LTR Retrotransposon | LINE | 100,696 | 25,997,014 | 3.49 |
|  | PLE | 4,354 | 2,052,977 | 0.28 |
|  | SINE | 3,221 | 706,909 | 0.09 |
|  | TRIM | 3,623 | 2,015,491 | 0.27 |
|  | unclassified | 958 | 201,950 | 0.03 |
| **Class II: DNA Transposon** | | 122,100 | 43,867,024 | 5.89 |
|  | Crypton | 1,633 | 1,507,492 | 0.2 |
|  | Helitron | 32,303 | 8,581,257 | 1.15 |
|  | MITE | 3,642 | 701,793 | 0.09 |
|  | Maverick | 4,712 | 1,645,595 | 0.22 |
|  | TIR | 75,189 | 30,714,319 | 4.13 |
|  | unclassified | 4,621 | 1,312,378 | 0.18 |
| **Potential Host Gene** |  | 68,543 | 14,843,825 | 1.99 |
| **SSR** |  | 956 | 746,541 | 0.1 |
| **Unknown** |  | 203,171 | 50,792,516 | 6.82 |
| **Total** |  | **1,270,594** | **472,243,039** | **63.43** |

DIRS: Dictyostelium intermediate repeat sequence; LARD: large retrotransposon derivative; LTR: long terminal repeat; LINE: long interspersed nuclear element; PLE: Penelope-like elements; SINE: short interspersed nuclear element; TRIM: terminal repeat retrotransposon in miniature; MITE: miniature inverted-repeat transposable element; TIR: terminal inverted repeat; SSR: simple sequence repeat.

**Table S13.** Statistic results of synteny within *R. molle* genome

| **Species** | **block number** | **match**  **numbers** | **total**  **gene number** | **1** | **2** | **3** | **4** | **5** |
| --- | --- | --- | --- | --- | --- | --- | --- | --- |
| *R. molle* | 162 | 1,228 | 2,215 | 2035 | 147 | 11 | 16 | 6 |

**Table S14.** Summary of the gene families among *R. molle* and ten other plant species

| ***Species*** | **Total**  **genes** | **Cluster genes** | **Total**  **families** | **Unique gene**  **families** | **Unique**  **genes** |
| --- | --- | --- | --- | --- | --- |
| *A. chinensis* | 33,044 | 30,808 | 17,022 | 250 | 592 |
| *A. thaliana* | 27,449 | 18,286 | 12,508 | 2,487 | 6,697 |
| *O. sativa* | 28,043 | 15,177 | 10,415 | 2,490 | 6,245 |
| *P. trichocarpa* | 31,305 | 29,606 | 16,364 | 837 | 3,044 |
| *P. veris* | 18,207 | 15,802 | 12,765 | 162 | 438 |
| *R. delavayi* | 32,938 | 28,568 | 20,692 | 256 | 594 |
| *R. molle* | 39,288 | 33,808 | 21,244 | 605 | 1,714 |
| *R. simsii* | 32,999 | 28,407 | 18,196 | 595 | 1,781 |
| *R. williamsianum* | 23,548 | 20,416 | 16,127 | 126 | 297 |
| *S. lycopersicum* | 25,423 | 24,115 | 19,211 | 209 | 774 |
| *S. tuberosum* | 28,209 | 26,575 | 19,419 | 361 | 1,307 |

**Table S17.** The summary of the TPS family in *R. molle* genome

| **Species** | **Genome**  **size (Mb)** | **Chromosome**  **number** | **TPS gene** | **TPS gene**  **density** | **TPS subfamily** | | | |
| --- | --- | --- | --- | --- | --- | --- | --- | --- |
|  |  |  | **number** |  | **a** | **b** | **c** | **e/f** |
| *R. molle* | 744.36 | 13 | 50 | 0.067 /Mb | 23 | 22 | 4 | 2 |

**Table S18.** The duplicated pairs of terpene synthases in *R. molle* genome

| **Tandem duplicated pairs** | | |  |  |  |
| --- | --- | --- | --- | --- | --- |
| Gene1 | Gene2 | Ka | Ks | Ka/Ks | Mya |
| RmTPS34 | RmTPS32 | 0.005412 | 0.034828 | 0.155405 | 2.487692 |
| RmTPS16 | RmTPS12 | 0.183788 | 0.605066 | 0.303748 | 43.21901 |
| RmTPS12 | RmTPS18 | 0.191054 | 0.647882 | 0.29489 | 46.27726 |
| RmTPS39 | RmTPS37 | 0.090435 | 0.248484 | 0.363945 | 17.74888 |
| RmTPS13 | RmTPS28 | 0.086142 | 0.112951 | 0.762645 | 8.067952 |
| RmTPS17 | RmTPS13 | 0.186575 | 0.605261 | 0.308255 | 43.23292 |
| RmTPS18 | RmTPS27 | 0.224196 | 0.679737 | 0.329828 | 48.55265 |
| RmTPS39 | RmTPS36 | 0.056769 | 0.123697 | 0.458935 | 8.835526 |
| RmTPS15 | RmTPS16 | 0.038355 | 0.124729 | 0.307509 | 8.909225 |
| RmTPS3 | RmTPS2 | 0.206535 | 0.34096 | 0.605747 | 24.35425 |
| RmTPS46 | RmTPS45 | 0.169844 | 0.334688 | 0.507469 | 23.90626 |
| RmTPS42 | RmTPS46 | 0.159308 | 0.342526 | 0.465098 | 24.46612 |
| RmTPS44 | RmTPS43 | 0.023486 | 0.065903 | 0.356373 | 4.707334 |
| RmTPS47 | RmTPS20 | 0.044173 | 0.07858 | 0.562141 | 5.612868 |
| **Segmental duplicated pairs** | | |  |  |  |
| Gene1 | Gene2 | Ka | Ks | Ka/Ks | Mya |
| RmTPS11 | RmTPS21 | 0.543794 | 2.581921 | 0.210616 | 184.423 |
| RmTPS1 | RmTPS3 | 0.242843 | 0.897277 | 0.270645 | 64.0912 |
| RmTPS41 | RmTPS45 | 0.69985 | 1.43721 | 0.48695 | 102.6578 |
| RmTPS41 | RmTPS21 | 0.69032 | 2.008757 | 0.34362 | 151.087 |
| RmTPS38 | RmTPS48 | 0.240413 | 0.58899 | 0.408177 | 42.07074 |
| RmTPS7 | RmTPS44 | 0.264946 | 0.855906 | 0.309551 | 61.13615 |
| RmTPS21 | RmTPS41 | 0.692898 | 1.232958 | 0.56198 | 88.06845 |

**Table S19.** The distribution of TPS tandem clusters in chromosomes

| **chromosome** | **Gene numbers** | **Range (kb)** | **Family** |
| --- | --- | --- | --- |
| LG12 | 5 | 80 | TPS-a |
| LG05 | 2 | 45 | TPS-a |
| LG05 | 6 | 230 | TPS-a |
| LG13 | 2 | 46.2 | TPS-a |
| LG11 | 2 | 23.8 | TPS-b |
| LG01 | 7 | 458 | TPS-b |
| LG05 | 2 | 47.8 | TPS-b |
| LG11 | 2 | 28.1 | TPS-c |

**Table S20.** All plasmids and strains used in this study

|  | **Genotype or characteristic** | **source** |
| --- | --- | --- |
| **Plasmids** |  |  |
| pESC-URA-RmTPS1 | *P_GAL10_-RmTPS1-T_ADH1_* | This study |
| pESC-URA-RmTPS2 | *P_GAL10_-RmTPS2-T_ADH1_* | This study |
| pESC-URA-RmTPS1-RmTPS5 | *P_GAL10_-RmTPS1-T_ADH1_ P_GAL1_-RmdiTPS5-T_CYC1_* | This study |
| pESC-URA-RmTPS2-RmTPS5 | *P_GAL10_-RmTPS2-T_ADH1_ P_GAL1_-RmdiTPS5-T_CYC1_* | This study |
| pESC-LEU-(B-E)-tHMG | *P_GAL1_*-*tHMG*-*T_CYC1_ P_GAL10_*-(*BTS1-ERG20*)-*T_ADH1_* | This study |
| **Strains** |  |  |
| INVSc1 | *MATa, his3Δ1, leu2, trp1-289, ura3-52 /MATα, his3Δ1, leu2, trp1-289, ura3-52* | Invitrogen |
| IN-0 | INVSc1 Δ*erg9* | This study |
| IN-1 | IN-0 with pESC-LEU-(B-E)-tHMG | This study |
| IN-2 | IN-1 with pESC-URA-RmTPS1 | This study |
| IN-3 | IN-1 with pESC-URA-RmTPS1 | This study |
| IN-4 | IN-1 with pESC-URA-RmTPS1-RmTPS5 | This study |
| IN-5 | IN-1 with pESC-URA-RmTPS2-RmTPS5 | This study |

**Table S24.** All primers used in this study

| **Primers** | **Sequences （5´→3´）** |
| --- | --- |
| **Primers for construction of *ERG9* deletion cassette** | |
| A | CAATAACCTTACCAATAACCGTCGCCC |
| B | CTAATTGTTGCAGCCTCTAACAACAC |
| C | TGAATCTAAGTGGAGAACAGGTGTG |
| D | GCGTACGAAGCTTCAGCTG CAGCTTCAAAGCTGCCTTCATCTCG |
| E | CAGATCCACTAGTGGCCTATGC AGTCTGCGCCAAATAACATAAAC |
| F | TAGTAAGCTAACCACTAATATAGCC |
| M-A | CAGCCAGTTTAGTCTGACCATCT |
| M-B | CGCAGACCGATACCAGGATCTTG |
| **Primers for cloning of BTS1, ERG20 and t-HMG from yeast genome** | |
| BTS1-F | ATGGAGGCCAAGATAGATGAGCTG |
| BTS1-R | TCACAATTCGGATAAGTGGTCTATT |
| ERG20-F | ATGGCTTCAGAAAAAGAAATTAG |
| ERG20-R | CTATTTGCTTCTCTTGTAAACTTTG |
| t-HMG-F | ATGGACCAATTGGTGAAAACTGAAG |
| t-HMG-R | TTATAATAATGCTGAGGTTTTACAG |
| **Primers for construction of the fusion of BTS1 and ERG20** | |
| BTS1(Fusion)-F | AAGGAAAAAAGCGGCCGCATGGAGGCCAAGATAGATGAGCTG |
| BTS1(Fusion)-R | TCACAATTCGGATAAGTGGTCTATTATATATAACAATTCGTCATGTAA |
| ERG20(Fusion)-F | AAGGAAAAAAGCGGCCGCATGGCTTCAGAAAAAGAAATTAGG |
| ERG20(Fusion)-R | GGACTAGTCTATTTGCTTCTCTTGTAAACTTTG |
| **Primers for RACE cloning of three diterpene synthases** | |
| RmTPS1-3**´** | AGCTTGAAGATGAAGATGCCGAGCACATG |
| RmTPS2-3**´** | ACAAGGGGATAATACTGGGCCCAGGACTTG |
| RmTPS5-3**´** | AGTTCTGCTACCGACGAGAGTGAACATTC |
| RmTPS5-5**´** | CTAATGTTGGCAGTGACTGGTCACTAGTCCA |
| **Primers for full-length cloning of three diterpene synthases** | |
| RmTPS1(FL)-F | ATGTCCTCTCATGATCTCTTCACCC |
| RmTPS1(FL)-R | AATTACTCTTTCAAAGAGTACTTTAGC |
| RmTPS2(FL)-F | ATGCGGTGTGAAATACCGCAC |
| RmTPS2(FL)-R | AATTACTGACTCAAATAGTACTTTGGC |
| RmTPS5(FL)-F | ATGTCTTTCATCCTCCCCACCAC |
| RmTPS5(FL)-R | TTATCCAGAGAGGGAAAGTGGTTCG |
| **Primers for plasmids construction** | |
| RmTPS1(P)-F | CGAATTCAACCCTCACTAAAGGGCATGTGCAAACCTAGTACAAA AGAAC |
| RmTPS1(P)-R | GTCATCCTTGTAATCCATCGATACTAAATTACTCTTTCAAAGAGTACTTTAG |
| RmTPS2(P)-F | CGAATTCAACCCTCACTAAAGGGCATGACGCAGAGTACATGGGATAACAACAAAAGAAAC |
| RmTPS2(P)-R | GTCATCCTTGTAATCCATCGATATCAAATTACTGACTCAAATAGTACTTTGGCAATG |
| RmTPS5(P)-F | ACGTACAGGAGAAAAACCCCGATCATGTCTTTCATCCTCCCCACCAC |
| RmTPS5(P)-R | GCTAGCCGCGGTACCATTATCCAGAGAGGGAAAGTGGTTCG |

The underlined sequences of D and E primers indicate the upstream and downstream homologous sequences of pUG6 loxP region, respectively.
