# Supplementary material for: Chromosome-scale genome assembly of Rhododendron molle provides insights into its evolution and terpenoid biosynthesis
Source: BMC Plant Biol. 2022 Jul 15;22:342. doi: 10.1186/s12870-022-03720-8 (PMC9284817; doi:10.1186/s12870-022-03720-8)
Supplement: Supplementary file 4 — Additional file 4. Methods S1. RNA sequencing and assembly. Methods S2. Gene duplication events and selective pressure analyses. Methods S3. Construction of CYPs phylogenetic tree and analysis of gene structure. Methods S4. Gas chromatographic-mass spectrometric analysis. Methods S5. Product purification and structure identification. [file 12870_2022_3720_MOESM4_ESM.docx]

**Methods S1.** RNA sequencing and assembly

Total RNA of mixed tissues (root, stem and leaf) was extracted using Trizol reagent, genomic DNA contamination was removed using the RNase-free DNase I. The integrity and quality of the RNA were evaluated using Agilent 2100 Bioanalyzer and NanoDrop spectrophotometer, respectively. We constructed the cDNA library using the NEBNext UltraRNA Library Prep Kit for Illumina (NEB), following standard protocol from the manufacturer. The constructed library was sequenced on Illumina HiSeq 2000 platform, generating 150 bp PE reads. Clean data were obtained after filtering out low-quality reads and adaptors, and assembled using Trinity [1] (v2.8.3) for subsequent genome annotation.

**Methods S2.** Gene duplication events and selective pressure analyses

We analyzed the tandem replication and segmental replication events in genes from the CYP and TPS families using MCScanX toolkits[2]. Two genes from the same CYP or TPS family located on the same chromosome and within 10 genes apart were regarded as tandem duplicated pair. We used the Circos program[3] to visualize both tandem duplicated and segmental regions within or among chromosomes for TPS genes. The bar plot showing physical locations of CYPs was drew using TBtools [4]. The nonsynonymous and synonymous nucleotide substitutions (*Ka* and *Ks*) value of duplicated gene pairs were generated using the add *Ka* and *Ks* to collinearity program in MCScanX package[2]. The timing of duplicated events for each gene pairs were further calculated using the formula T = Ks/2λ (λ = 7 × 10^−9^).

**Methods S3.** Construction of CYPs phylogenetic tree and analysis of gene structure

The 294 RmCYP protein sequences were aligned with *A. thaliana* CYPs by MUSLE[5] using default parameters. The maximum-likelihood (ML) phylogenetic tree was constructed using FastTree (v2.1.7)[6] with JTT substitution model and the robustness/accuracy of tree topology was evaluated by 1000 bootstrap replicates. Interactive Tree of Life (iTOL)[7] was used to visualized the ML tree. The phylogenetic tree of CYP71 candidates from *A. thaliana*, *C. sativus*, *R. simsii*, *R. williamsianum*, *R. delavayi* and *R. molle* was constructed using the same method. The MEME web platform (http://meme-suite.org/tools/meme) was employed to detected conserved protein motifs among RmCYPs with the following parameters: motifs number as 30, motif width range as 8 to 30.The GFF3 (Generic Feature Format version 3) annotation file of *R. molle* genome was analyzed by TBtools [4] for gene structure visualization, the intron phases distribution of each CYP genes were also determined.

**Methods S4.** Gas chromatographic-mass spectrometric analysis

GC analyses were performed on a Shimadzu GCMS-QP-2020 using SH-Rxi-5SilMS column (30m*0.25μm*0.25mm), the GC was coupled to a triple quadrupole detector. Helium was used as the carrier gas with 1.6mL /min flow rate. The injection volume was 1μL at 280°C in spitless mode. For diterpene analysis, the GC program was as follow: 50°C, hold for 2 min, ramp at rate 10°C /min to 280°C, and hold for 15 min. MS data in the range from 60 to 650 mass-to-charge ratio (m/z) were collected after 3 min solvent delay.

**Methods S5.** Product purification and structure identification

8 L fermentation including both cell pellets and the culture media was extract with twice volume hexane, crude extracts were separated by preparative high performance liquid chromatography equipped with Shimadazu LC-6AD instrument (SPD-20A and RID-10A detectors) (Shimadzu Corporation, Kyoto, Japan). A YMC ODS column (C18, 250 × 20 mm, 5μm) was used with the following two programs (primary preparation: 0-50 min A: 90% CH_3_OH B: 10% ddH2O, 3.5 ml/min; second preparation: 0-50 min A: 85% ACN B: 15% ddH2O, 3.5 ml/min). The purified products were dissolved in deuterated pyridine (C5D5N; Sigma-Aldrich) containing tetramethylsilane (TMS). NMR spectra were recorded at 25 °C on a INOVA 600 NMR spectrometer (Varian, California, USA).

**Supporting References**

1. Grabherr MG, Haas BJ, Yassour M, Levin JZ, Thompson DA, Amit I, et al. Full-length transcriptome assembly from RNA-Seq data without a reference genome. Nature Biotechnology. 2011;29(7):644-52.

2. Wang Y, Tang H, Debarry JD, Tan X, Li J, Wang X, et al. MCScanX: a toolkit for detection and evolutionary analysis of gene synteny and collinearity. Nucleic acids research. 2012;40(7):e49-e.

3. Krzywinski M, Schein J, Birol I, Connors J, Gascoyne R, Horsman D, et al. Circos: an information aesthetic for comparative genomics. Genome research. 2009;19(9):1639-45.

4. Chen C, Chen H, Zhang Y, Thomas HR, Frank MH, He Y, et al. TBtools: an integrative toolkit developed for interactive analyses of big biological data. Molecular plant. 2020;13(8):1194-202.

5. Edgar RC. MUSCLE: a multiple sequence alignment method with reduced time and space complexity. BMC bioinformatics. 2004;5(1):1-19.

6. Price MN, Dehal PS, Arkin AP. FastTree 2–approximately maximum-likelihood trees for large alignments. PloS one. 2010;5(3):e9490.

7. Letunic I, Bork P. Interactive tree of life (iTOL) v3: an online tool for the display and annotation of phylogenetic and other trees. Nucleic acids research. 2016;44(W1):W242-W5.
